# A Blended Web-Based Gaming Intervention on Changes in Physical Activity for Overweight and Obese Employees: Influence and Usage in an Experimental Pilot Study

**DOI:** 10.2196/games.6421

**Published:** 2017-04-03

**Authors:** Tessa A Kouwenhoven-Pasmooij, Suzan JW Robroek, Sui Wai Ling, Joost van Rosmalen, Elisabeth FC van Rossum, Alex Burdorf, MG Myriam Hunink

**Affiliations:** ^1^ Department of Epidemiology Erasmus MC, University Medical Center Rotterdam Rotterdam Netherlands; ^2^ Department of Public Health Erasmus MC, University Medical Center Rotterdam Rotterdam Netherlands; ^3^ Department of Occupational Health Erasmus MC, University Medical Center Rotterdam Rotterdam Netherlands; ^4^ Department of Biostatistics Erasmus MC, University Medical Center Rotterdam Rotterdam Netherlands; ^5^ Department of Internal Medicine Erasmus MC, University Medical Center Rotterdam Rotterdam Netherlands; ^6^ Obesity Center CGG Rotterdam Netherlands; ^7^ Department of Radiology Erasmus MC, University Medical Center Rotterdam Rotterdam Netherlands; ^8^ Harvard T.H. Chan School of Public Health Center for Health Decision Sciences Harvard University Boston, MA United States

**Keywords:** eHealth, gamification, physical activity, fitness tracker, body mass index, engagement, social support, blended care

## Abstract

**Background:**

Addressing the obesity epidemic requires the development of effective interventions aimed at increasing physical activity (PA). eHealth interventions with the use of accelerometers and gaming elements, such as rewarding or social bonding, seem promising. These eHealth elements, blended with face-to-face contacts, have the potential to help people adopt and maintain a physically active lifestyle.

**Objective:**

The aim of this study was to assess the influence and usage of a blended Web-based gaming intervention on PA, body mass index (BMI), and waist circumference among overweight and obese employees.

**Methods:**

In an uncontrolled before-after study, we observed 52 health care employees with BMI more than 25 kg/m^2^, who were recruited via the company’s intranet and who voluntarily participated in a 23-week Web-based gaming intervention, supplemented (blended) with non-eHealth components. These non-eHealth components were an individual session with an occupational health physician involving motivational interviewing and 5 multidisciplinary group sessions. The game was played by teams in 5 time periods, aiming to gain points by being physically active, as measured by an accelerometer. Data were collected in 2014 and 2015. Primary outcome was PA, defined as length of time at MET (metabolic equivalent task) ≥3, as measured by the accelerometer during the game. Secondary outcomes were reductions in BMI and waist circumference, measured at baseline and 10 and 23 weeks after the start of the program. Gaming elements such as “compliance” with the game (ie, days of accelerometer wear), “engagement” with the game (ie, frequency of reaching a personal monthly target), and “eHealth teams” (ie, social influence of eHealth teams) were measured as potential determinants of the outcomes. Linear mixed models were used to evaluate the effects on all outcome measures.

**Results:**

The mean age of participants was 48.1 years; most participants were female (42/51, 82%). The mean PA was 86 minutes per day, ranging from 6.5 to 223 minutes, which was on average 26.2 minutes per day more than self-reported PA at baseline and remained fairly constant during the game. Mean BMI was reduced by 1.87 kg/m^2^ (5.6%) and waist circumference by 5.6 cm (4.8%). The univariable model showed that compliance, engagement, and eHealth team were significantly associated with more PA, which remained significant for eHealth team in the multivariable model.

**Conclusions:**

This blended Web-based gaming intervention was beneficial for overweight workers in becoming physically active above the recommended activity levels during the entire intervention period, and a favorable influence on BMI and waist circumference was observed. Promising components in the intervention, and thus targets for upscaling, are eHealth teams and engagement with the game. Broader implementation and long-term follow-up can provide insights into the sustainable effects on PA and weight loss and into who benefits the most from this approach.

## Introduction

Worldwide, 2.1 billion individuals are overweight or obese and the prevalence keeps increasing [1]. This a major burden for not only individual health but also health care and societal costs [2]. Physical activity is important to enhance weight loss and for the prevention of weight gain, reducing the risks of serious health problems such as cardiovascular disease, cancer, diabetes, osteoarthritis, and depression [3,4]. Adherence to physical activity recommendations among obese individuals is poor [5,6], creating an urgent need for a scalable, effective, and sustainable approach to enhance physical activity in the prevention and treatment of obesity. Although eHealth has this potential, attrition rates in eHealth programs are high [7,8], which means that sustainable behavior change may require a more intense approach [9]. The most promising approach for promoting healthy behavior in an efficient manner seems to be the combined use of successful eHealth components and non-eHealth components [10].

The eHealth components that have been shown to be promising elements of a successful Web-based health intervention are use of accelerometer or activity tracker [11] and gamification [12]. Accelerometers monitor the level of physical activity, which plays a critical role in reducing health risks and improving body composition [13-16] and is essential for long-term weight management in overweight and obese individuals [17]. There is a growing availability of such “quantified self” devices, which objectively measure an individual’s level of physical activity by means of the total amount, intensity, duration, and frequency of physical activities. In addition to objective registration of the level of physical activity, using an accelerometer can raise the individual’s awareness of his or her activity level [18] and consequently increase the level of physical activity [19,20]. Gamification is an emerging field and has shown to be promising, achieving its effectiveness by rewarding, social bonding, and making the health intervention fun to engage in [12], which is in common with proven health behavior change approaches [21,22]. Despite the advantages of a broad reach and easy accessibility [23], eHealth-only approaches tend to suffer from high attrition and dropout rates [8], which should be prevented if aiming for a sustainable lifestyle change.

Apart from eHealth, direct human contact by way of counseling can be an important component in lifestyle behavior programs. Motivational interviewing is a suitable counseling technique to improve exercise adherence [24] and weight loss [25,26], taking into account a patient’s readiness to make lifestyle changes as well as for planning and goal setting. A recent review suggested that direct human contact may help intensify the effect of eHealth technologies [10]. There is a lack of evidence on the effectiveness and usage of programs in which eHealth and non-eHealth components are blended for optimal effectiveness, reach, adherence, and costs.

Aiming for a both effective and efficient intervention with blended usage of eHealth components and non-eHealth components, we developed our program and implemented it in a pilot setting. The results of this pilot study will inform us whether broader implementation with longer follow-up is useful for this target population. Therefore, the aims of this study were to analyze the sustainability of physical activity during the game and to assess changes in body mass index (BMI) and waist circumference. In addition, we aimed to assess the influence of compliance, engagement, and eHealth teams on these outcomes.

## Methods

### Study Design and Population

This uncontrolled, before-after pilot study evaluates a blended Web-based gaming intervention for overweight and obese employees to become more physically active and adopt a healthy diet in a way that suits their personal preferences and abilities and, ultimately, to lose weight. The program was developed and implemented by the occupational health center of the Erasmus MC, University Medical Center in Rotterdam to improve the vitality and well-being of its overweight and obese employees. The main idea was developed and tested in 2010 and upgraded to the current version in 2013, which was tested by a test group before implementation in our study population. Key objectives of this program are to encourage overweight employees to become more physically active and adopt a healthy diet in a way that suits their personal preferences and abilities and, ultimately, to lose weight. The program consists of a face-to-face individual session with an occupational health physician, 5 group sessions, and a 20-week movement game that is played in real life, using accelerometers to measure physical activity.

Participants were recruited by memos on the company’s intranet in December 2013 and in September 2014 and were selected based on being overweight or obese (BMI ≥25 kg/m^2^) or having a large waist circumference (≥102 cm for men and ≥88 cm for women) and being motivated to change their lifestyle. Because of the Web-based approach, affinity with computers was desirable, but only computer accessibility was required. Excluded from participation were employees who (1) were using medication with weight gain being a side effect, (2) were unable to be physically active, (3) were currently pregnant or breastfeeding, or had the wish to be pregnant within 23 weeks, (4) did not speak Dutch, or (5) needed an intervention for an additional problem (alcohol intervention, thyroid regulation). Selection for the program took place during a 30-minute individual session with the occupational health physician.

Participation in the program was voluntary and no individual information was shared with anyone, especially not with the employer or direct supervisor. The program was free of charge for the first 24 applicants because this was covered by a grant. When the program was offered half a year later to an additional 28 applicants, the program content remained identical, but a participation fee of €450 was introduced to cover the workshop and the accelerometer. The study protocol was approved by the Medical Ethics Committee of the Erasmus University Medical Center (registration numbers MEC-2015-134 for overweight participants and MEC-2012-257 for obese participants), and signed informed consent forms were obtained from all participants. Although this is not a randomized controlled trial, reporting of the study was performed according to the CONSORT-EHEALTH (Consolidated Standards of Reporting Trials of Electronic and Mobile Health Applications and Online Telehealth) standards where applicable [27]. See Multimedia Appendix 1 for the CONSORT-EHEALTH checklist.

### Intervention

Gaming components of social bonding, rewarding, and competition are included throughout the program, which is offered in a combination of eHealth and face-to-face care (non-eHealth), that is, a blended intervention.

#### Non-eHealth

##### Session With Occupational Health Physician

During this session, motivational interviewing was used to determine motivation to make lifestyle changes and to start individual planning and goal setting [28]. After being selected, participants received a confirmation letter stating the start date of the program and instruction on how to purchase the obligatory accelerometer.

##### Group Sessions

Group sessions took place in the 1st, 2nd, 3rd, 4th, and 12th week of the program and lasted 2.5 to 3 hours each. Each group consisted of a maximum of 20 participants. Because obesity requires a multidisciplinary approach [29], the sessions were alternately given by a physician, a dietician, a physical therapist, and a psychologist. During the group sessions, participants (1) were educated on the health risks of obesity and the benefits of a healthy lifestyle, including physical activity, diet, alcohol consumption, and relaxation; (2) were guided in individual goal setting and planning and challenged to makes choices that would be sustainable in regard to personal preferences and social context, with the aim to increase physical activity and lower caloric intake; and (3) received explanation on the movement game and on the use of the accelerometer. Social networking with fellow group members was stimulated during all sessions.

#### eHealth: Movement Game

**Figure 1:**
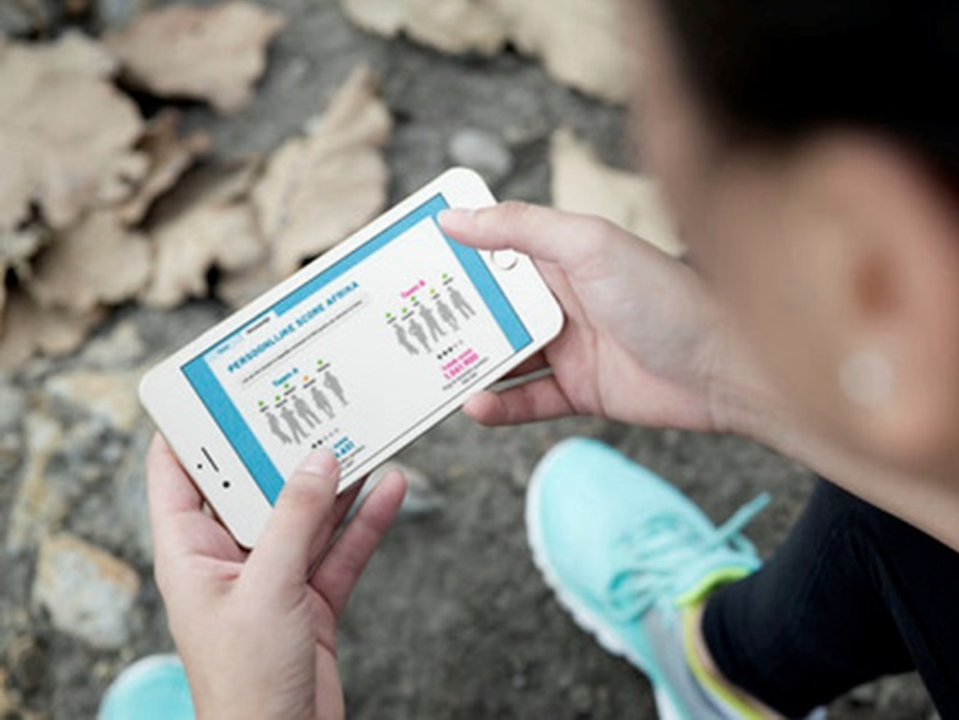
Screenshot of the movement-game.

The movement game is a Web-based tour around the world, which is played by being physically active in the “real world.” Touring the world takes 20 weeks, and every 4 weeks the tour crosses another continent (Europe, North America, Asia, Australia, Africa). The game was played by 2 competing teams aiming to win the continent by scoring the most “movement points.” An independent “game coach” randomly divided the participants of one program into 2 eHealth teams, which he announced during the third group session along with the rules of the game. Every team member strove to reach his or her personal target, which was set before the first continent by the physical therapist. Movement points were granted according to the duration and intensity of physical activity, which was registered by an accelerometer. Players were asked to upload the accelerometer data into the Web-based movement game via a USB connection at least once a week and were educated on the Dutch norm of physical activity, which is being physically active at least 5 times a week for 30 minutes (21.4 minutes/day) at MET (metabolic equivalent task) 3 or higher, and on the “fit norm,” which is physical activity at least 3 times a week for 20 minutes (8.6 minutes/day) at MET 6 or higher [30]. If a personal target was reached within a continent, a written advice for raising the target for the next continent appeared on the personal webpage. Participants could visually monitor their progression toward their individual targets and against the other team at any time, both on a desktop computer and on a mobile phone. Figure 1 shows a screenshot of the gaming intervention. Multimedia Appendix 2 provides additional screenshots of the movement game, illustrating the competition.

Registration of physical activity was performed by the Activ8 system (Remedy Distribution Limited, Valkenswaard, The Netherlands), which is a small triaxial accelerometer that is worn in the pocket of any pants or with a leg strap on the upper leg [31]. The Activ8 output was tested against video analysis, and sensitivity scores of postures and movements ranged from 81% to 98%. The game coach handed out instructions for installing the Activ8 software on the computer and assisted if necessary. A critical requirement for sufficient valid functioning is wearing the device in the correct position and without (excessive) tilting; this was specifically instructed by the game coach in our study. During the game, the Activ8 device needed to be worn at all times, except during swimming and sleeping. Because swimming was not registered by the Activ8 device, the number of swim minutes could be filled out manually on the game’s webpage.

Every 2 weeks an automatic email was sent to the participants, providing general information on multiple lifestyle aspects related to the upcoming continent. If participants failed to log on to the game’s website for more than 2 weeks, an email reminder was sent by the game coach. An online social network was provided by the game by means of a digital forum page. Written messages, as well as responses to these messages, could be posted by the participants or the game coach. The game coach could be consulted every working day at the occupational health center and was the same person throughout the program.

Awards could be won both individually and as teams. Virtual bronze, silver, and gold medals would appear for every individual achieving 80%, 90%, or 100% of their individual target within a continent. In addition, after completion of a 4-week continent, 1 individual player and all members of the winning team received tangible gifts related to a healthy lifestyle, such as a sports towel or a water bottle. The individual winner was selected by the game coach based on having collected the most movement points, having made the most progress, or showing the best team spirit on the forum. The game coach announced the continent winners by a message on the Web-based forum and granted the awards personally.

Although uploading of movement points by the accelerometer could only be done using a desktop computer, all other aspects of the game were accessible by mobile phone as well. Confidentiality of users was ensured by using only first names in the game. To ensure security of content and users, the game used password-protected accounts, encrypted password storage, encrypted log-in details, and secure external servers. During the program, no interfering bug fixing was needed.

### Measurements

Collection of baseline characteristics (age, sex, level of education, shift work, working hours per week) was done during the individual selection session with the company’s occupational health physician (TK), between February 2014 and July 2015. Educational level was categorized into 2 categories (low or medium and high) according to the Dutch educational system.

Participants with BMI ≥30 kg/m^2^ were additionally seen by the specialist for internal diseases and endocrinology (EvR) of the Obesity Center CGG (“Centrum Gezond Gewicht”) of the Erasmus Medical Center of Rotterdam to ensure appropriate treatment of underlying or complicating diseases. Costs were covered by the health insurance company, with the exception of an individual’s deductible. Because participants with BMI ≥30 kg/m^2^ were remeasured at 10 weeks by this specialist, we added this 10-week measurement to participants with BMI 25-30 kg/m^2^ in groups 3 and 4 in order for the measurements to be identical for all BMI categories.

### Primary Outcome: Average Physical Activity per Day (Average MVPA)

The Activ8 accelerometer provided information on the number of seconds spent at a certain MET level, which was collected by the supportive information and technology company (ICT) at the end of the 20-week movement game. We categorized physical activity into time spent in moderate physical activity (MPA) and time spent in vigorous physical activity (VPA), expressed in MET-hours. The cutoff energy levels used for this study were ≥3 to 6 METs for MPA and ≥6 METs for VPA. The cutoffs are based on the Dutch recommendations for healthy behavior [30]. Moderate to vigorous physical activity (MVPA) was the sum of MPA and VPA, also expressed in MET-hours. The accelerometer had to register ≥10 hours per day of activity at >1 MET to count as a “valid day.” We registered a “nonvalid day” when no more than 10 hours of activity was registered or when there was no registration at all because the battery ran out, because we interpreted this as nonusage of the device. We calculated the average MVPA in MET-hours by dividing MVPA during the game by the number of valid days.

### Secondary Outcomes: Body Mass Index and Waist Circumference

For weight measurements, the occupational health physician used the scale that was available for daily practice (Inventum PW705BG (Arnhem, The Netherlands)), which is calibrated once a year and remained the same throughout the study period. For calculating BMI in kg/m^2^, body height was self-reported at baseline, which differed less than 1% from objective measures at 10 and 23 weeks, and the value was kept the same in all BMI calculations. Waist circumference was always measured by the same occupational health physician and was measured halfway between the lower rib and the iliac crest, as is advised by the Dutch obesity recommendations for general practitioners [32]. Both measurements were done at baseline and 10 and 23 weeks after baseline. Delta BMI and delta waist circumference were used as outcome parameters, which were the measurements at baseline minus those at 10 and 23 weeks.

### Determinants: Compliance, Engagement, eHealth Team, and Other

#### Compliance

A program-specific demand was used as behavioral measure of compliance, which was the percentage of days with more than 10 hours of accelerometer wear during the 20-week game (ie, accelerometer wear).

#### Engagement

Engagement was measured as the number of times at least 100% of the personal target level was reached (ranging from 0 to 5) and categorized into ≤3 times and 4 or 5 times.

#### eHealth Teams

All participants were randomly assigned to an eHealth team (8 teams in total) for social influencing. For the purpose of analyses, we categorized teams into dummy numbers 1 to 8.

#### Other Measures of Usage

To further assess usage of the game, we measured the number of log-ins on the game website and the number of messages posted on the forum.

### Data Analysis

Descriptive statistics were used to present the baseline characteristics of the study population. We excluded data of 1 participant because of pregnancy. The primary outcome measure was average MVPA (time-weighted area under the curve of the MET level) during the 20-week period of the game. Secondary outcomes were reductions in BMI and waist circumference versus baseline. Determinants were compliance to and engagement with the game and team effects.

In univariable linear regression analyses we investigated the association between age, sex, educational level, BMI at baseline, working hours per week, shift work, eHealth team, compliance (accelerometer wear), engagement (number of times the target level was reached), and other measures of usage (number of log-ins and messages on the forum) as independent variables and the average amount of MVPA per day as the dependent variable. To compare eHealth teams, we chose the team with the lowest average MVPA as the reference category. We log-transformed MVPA to create an approximately normal distribution of our outcome variable.

Univariable and multivariable analyses were performed using linear mixed models to account for the within-subject correlations due to intrateam effects and, in the case of BMI and waist circumference, for repeated measurements. The average amount of MVPA per day was used as outcome measure of the multivariable analyses, and the reductions in BMI and waist circumference at 10 and 23 weeks versus baseline were outcome measures of both univariable and multivariable analyses. We evaluated multiple models by combining different determinants in each model, aiming to get insight into the (combination of) independent variables with the most effect on the outcomes. The independent variables in separate and combined models were accelerometer wear as a measure of compliance and the number of times the individual target was reached as a measure of engagement; in the linear mixed models for the change in BMI and waist circumference during the intervention, we added the average amount of MVPA during the game and changes in time (10 and 23 weeks). All models were further adjusted for sex, age, and BMI at baseline. The variances between eHealth teams were included as random effects. A random intercept was included to account for the within-subject correlations. Collinearity between independent variables was assessed by calculation of the variance inflation factors. We considered including interaction effects between each independent variable and time, but this was not necessary because tests showed no significant interaction effects.

All statistical tests were two-sided and used a significance level of .05. All statistical analyses were performed using IBM SPSS version 22 (IBM Corporation).

## Results

**Figure 2:**
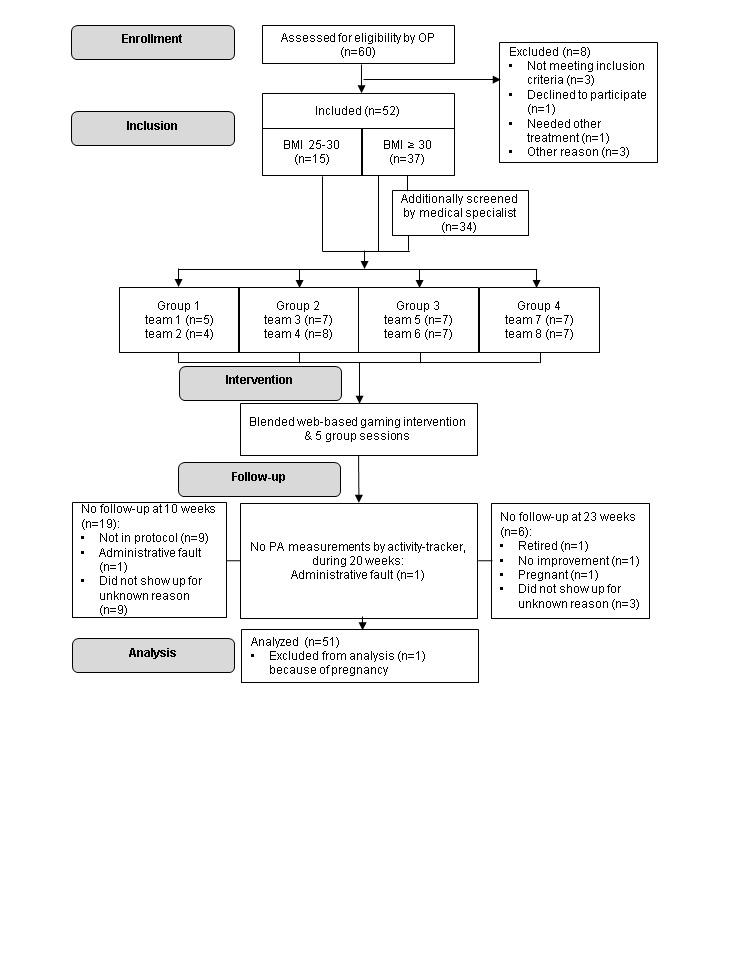
Flow of participants. BMI: body mass index; OP: occupational health physician; PA: physical activity.

In total, 52 employees participated in this program, of whom 1 participant was excluded from analyses because of pregnancy. Figure 2 shows the flow of participants in the program, including the number of participants, the grouping, and the program flow over time. Of the participants with BMI ≥30 kg/m^2^, 3 were not additionally screened at study inclusion by the specialist for internal diseases and endocrinology because of personal choices.

Baseline characteristics for all study participants are provided in Table 1. The mean age of the participants was 48.1 years, ranging from 29 to 65 years, and 69% received higher education. The majority of participants were female (42/51, 82%).

**Table 1:** Baseline characteristics of participants (n=51).

Characteristics		n (%)	Mean (SD)
**Sex**			
	Men	9 (18)	
	Women	42 (82)	
Age, years			48.1 (9.2)
**Educational level** (n=45)			
	Low or medium	14 (31)	
	High	31 (69)	
Insufficient physical activity^a^ (n=42)		31 (74)	
Weight, kg			96.3 (16.9)
**Waist circumference, cm**			
	Men		124.3 (14.59)
	Women		109.7 (10.8)
**Body mass index, kg/m^2^**			32.7 (5.1)
	25-30	15 (29)	27.2 (0.9)
	≥30	36 (71)	35 (4.4)
**Work**			
	Hours/week		30.8 (7.1)
	Shift work^b^	10 (20)	

^a^Defined as no adherence to the Dutch guideline at baseline.

^b^Evening or night shifts.

**Figure 3:**
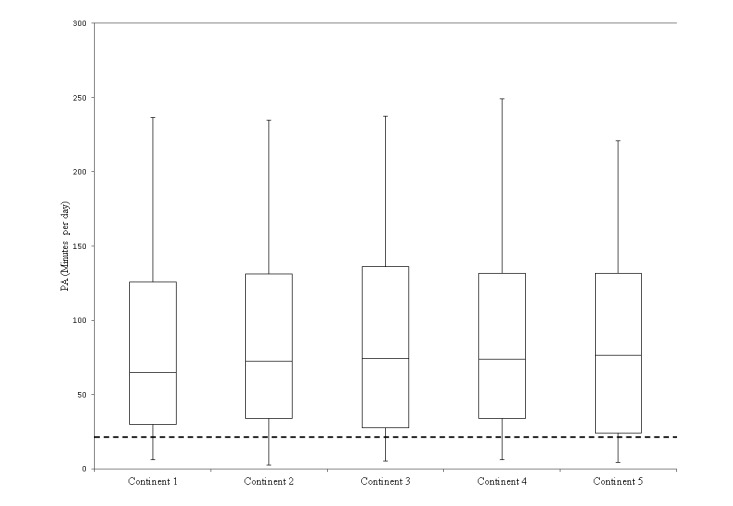
Box plot showing moderate to vigorous physical activity in minutes per day in all 5 continents of the game. The dashed line marks physical activity (PA; metabolic equivalent task or MET≥3) for 30 minutes at least 5 times a week (=150 minutes per week, which is on average 21.4 minutes per day). The top and bottom borders of the box mark the 75th and 25th percentiles; the horizontal line in the middle indicates the median. The whiskers mark the lowest and highest scores.

Figure 3 shows that the average MVPA remained fairly constant during the entire game and that the Dutch norm of physical activity was met by every individual in each continent, which is high as opposed to 26% at baseline based on self-reported data. The “fit norm” was not met by 90% of the participants (data not shown). The average MVPA during the game was 7.08 MET-hours, ranging from 0.5 to 18.89 MET-hours.

Table 2 shows that sex, age, level of education, BMI, and waist circumference at baseline as well as work parameters were not significantly associated with the average MVPA. Several elements of the game seem to be associated with a higher level of MVPA. After inversion of the log-transformed MVPA, the eHealth teams showed a 7.9-fold difference in increase in average MVPA (95% CI 4.2-14.8), illustrating the large variability in improvement in MVPA across teams. The average MVPA of teams ranged from 1.9 to 13.3 MET-hours per day. More compliance was also significantly associated with an increase in average MVPA. For example, 20 more days of wear would mean an increase of 35% of MVPA (95% CI 2%-79%) on the days the accelerometer was worn. On average, the accelerometer was worn for more than 10 hours per day on 89% of the available days, ranging from 44% to 100%. This percentage was above 80% in all 5 continents of the game. Compared with low engagement during the game (ie, infrequently reaching individual targets), there is an absolute gain in MVPA of 2.8-fold relative increase (95% CI 1.7-4.6) when being highly engaged. The individual target of physical activity was reached at the most 3 times by 32 (63%) participants and more than 3 times by 19 participants (37%).

**Table 2:** Association between baseline characteristics and program usage and physical activity (n=51): univariable linear regression analyses.

Characteristics		Moderate to vigorous physical activity^a^	
		Β (95% CI)	*P* value^b^
**Sex**			
	Women	Reference	
	Men	0.38 (−0.35 to 1.10)	.30
Age, years		0.01 (−0.03 to 0.04)	.76
**Educational level**			
	Low or medium	Reference	
	High	0.44 (−0.20 to 1.08)	.18
Body mass index, kg/m^2^		−0.02 (−0.07 to 0.04)	.53
Waist circumference, cm		0.10 (−0.01 to 0.03)	.36
Work, hours/week		−0.02 (−0.07 to 0.02)	.26
**Shift work**			
	Yes	Reference	
	No	0.23 (−0.50 to 0.96)	.53
**Characteristics of program usage**			
eHealth team (1-8)			
	Team 1	2.06 (1.43 to 2.70)	<.001
	Team 2	2.04 (1.37 to 2.72)	<.001
	Team 3	2.06 (1.46 to 2.66)	<.001
	Team 4	1.72 (1.14 to 2.29)	<.001
	Team 5	1.01 (0.43 to 1.59)	.001
	Team 6	0.09 (−0.48 to 0.67)	.75
	Team 7	0.45 (−0.13 to 1.02)	.13
	Team 8	Reference	
Compliance: accelerometer wear^c^		0.02 (0.00 to 0.04)	.04
Engagement			
	≤3 times target reached	Reference	
	4 or 5 times target reached	1.03 (0.52 to 1.53)	<.001
Number of log-ins		−0.00 (−0.01 to 0.01)	.74
Number of messages		0.01 (0.00 to 0.02)	.04

^a^Log-transformed average moderate to vigorous physical activity (metabolic equivalent task or MET>3), in MET hours.

^b^Statistical significance was defined as *P*<.05.

^c^Percentage of days with >10 hours of physical activity registration.

**Figure 4:**
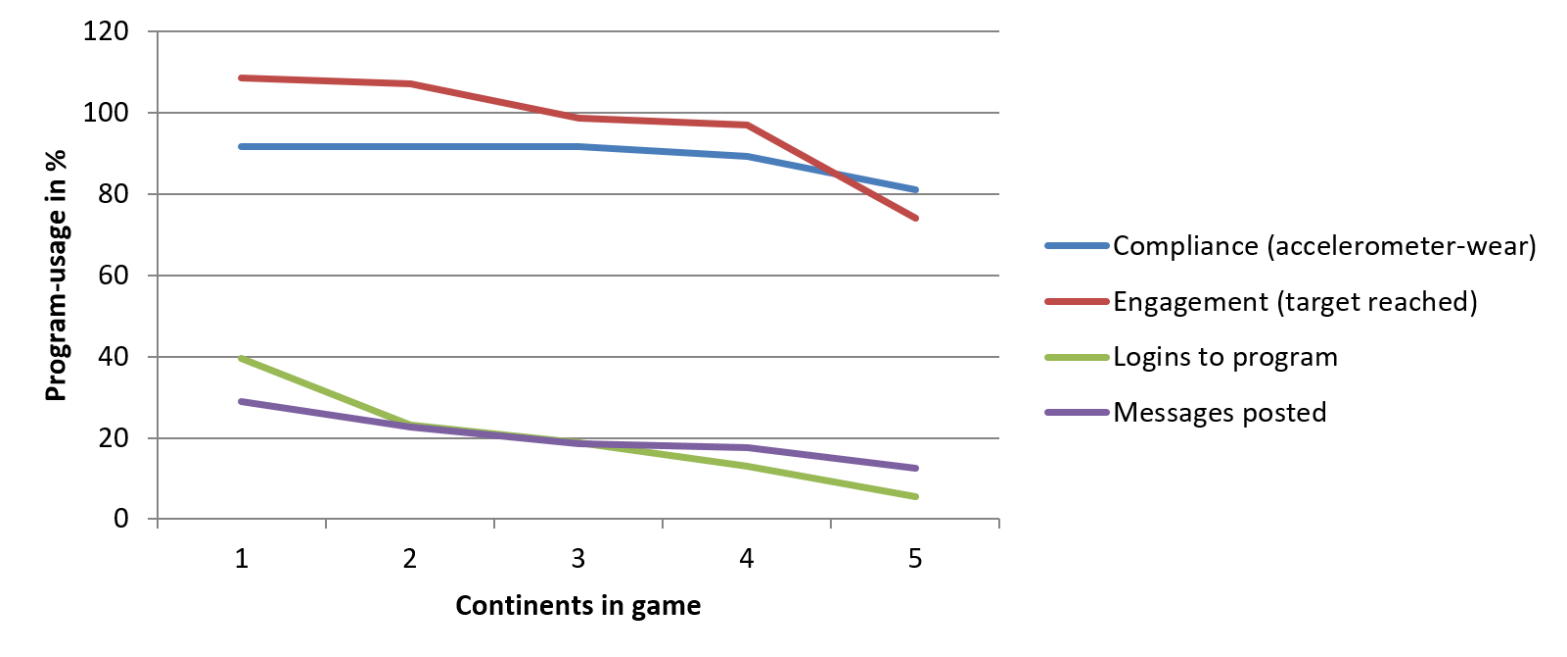
Attrition curve: program usage in the continents of the movement game. Compliance (accelerometer wear) is expressed as the average number of days with at least 10 hours of physical activity registration at >1 MET (metabolic equivalent task). Engagement (target reached) is expressed as physical activity registered by the accelerometer divided by the individual target level of physical activity within a certain continent. Log-ins to the program are expressed as the number of online log-ins within a certain continent divided by the number of online log-ins during the entire game. Messages posted are expressed as the number of messages posted on the Web-based forum within a certain continent divided by the number of messages posted during the entire game.

The attrition curve in Figure 4 shows a decrease in compliance and engagement and other measures of usage toward the end of the game, although the average MVPA remained fairly constant. A total of 4 participants showed no accelerometer wear in the last continent. Reasons for no uploads were vacation abroad for 2 participants, a lost device for 1 participant, and a lack of motivation for another participant. We note that some teams switched to alternative social media in the last continent, which may explain the decrease in messages on our forum.

Table 3 suggests that team membership has a fairly robust effect on average MVPA, because the standard deviation of the random effect of eHealth team remains similar in models 3 through 6 and because 0.45 (0.99 in model 2 minus 0.54 in model 3) of the variance between participants was explained by eHealth team. The heterogeneity between eHealth teams is presented as the standard deviation of the normally distributed random effects of eHealth teams for the log-transformed MVPA value. The value of 0.88 for this standard deviation in model 3 implies a 3.7-fold relative difference in average MVPA between two teams randomly chosen from the population, thus suggesting large differences between teams that could not be explained by age, sex, or BMI at baseline, nor were they additionally affected by compliance or engagement. The variance inflation factor did not exceed 1.5 for any independent variable, which indicates that there was no multicollinearity problem.

**Table 3:** Multivariable association between baseline characteristics and program usage and physical activity (n=51): separate models using linear mixed models.

Characteristics of program usage: models			Moderate to vigorous physical activity^a^		
			Sources of variance, SD^b^ (95% CI)		Β (95% CI)
Model: description	Independent variables (additional)	Categories	Between eHealth teams	Between participants	
Model 1: raw			N/A^c^	0.98 (0.80 to 1.19)	N/A
Model 2: baseline characteristics			N/A	0.99 (0.81 to 1.22)	
	Age				0.01 (−0.03 to 0.04)
	Sex	Women			Reference
		Men			0.43 (−0.33 to 1.19)
	BMI^d^ at baseline				−0.02 (−0.08 to 0.04)
Model 3: model 2 + eHealth team			0.88 (0.51 to 1.55)	0.54 (0.43 to 0.67)	
Model 4: model 3 + compliance			0.86 (0.49 to 1.52)	0.55 (0.44 to 0.69)	
	Compliance^e^	<85%			Reference
		85%-95%			0.15 (−0.34 to 0.65)
		≥95%			0.17 (−0.31 to 0.64)
Model 5: model 3 + engagement			0.83 (0.46 to 1.50)	0.54 (0.43 to 0.68)	
	Engagement^f^	≤3			Reference
		4 or 5			0.19 (−0.25 to 0.64)
Model 6: model 3 + compliance + engagement			0.82 (0.45 to 1.48)	0.56 (0.44 to 0.70)	
	Compliance	<85%			Reference
		85%-95%			0.17 (−0.34 to 0.67)
		≥95%			0.06 (−0.50 to 0.62)
	Engagement	≤3			Reference
		4 or 5			0.17 (−0.32 to 0.66)

^a^Log-transformed average moderate to vigorous physical activity (metabolic equivalent task or MET>3), in MET hours.

^b^SD is the standard deviation of the random effect between teams or between participants.

^c^N/A: not applicable.

^d^BMI: body mass index.

^e^Compliance is expressed as percentage of days with >10 hours of physical activity registration (accelerometer wear).

^f^Engagement is expressed as the number of times at least 100% of the target was reached (1-5).

**Figure 5:**
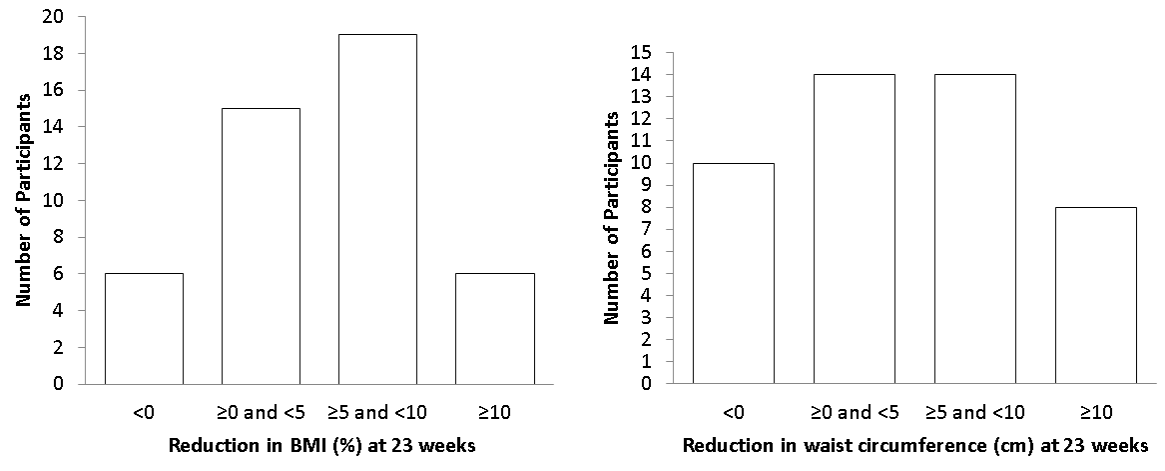
Reduction in weight and waist circumference of eHealth program participants, at 23 weeks versus baseline, in categories. BMI: body mass index.

Figure 5 shows the categories of reductions in BMI and waist circumference after 23 weeks. The mean BMI was reduced by 1.87 kg/m^2^ (range -8.7 to 2.4 kg/m^2^) during the program, corresponding to 5.6% (range -20.2% to 7.6%), and the mean waist circumference was reduced by 5.6 cm (range −4.5 to 23 cm). Univariable analysis showed significantly more reductions in BMI and waist circumference (BMI: B 0.12, 95% CI 0.04-0.20; waist circumference: B 0.22, 95% CI 0.09-0.36) when BMI and waist circumference values were higher at the start of the program (Multimedia Appendix 3).

Model 5 in Table 4 shows that more engagement was the only component associated with reductions in BMI (B 1.23, 95% CI 0.17-2.29) and waist circumference (Multimedia Appendix 4; B 4.44, 95% CI 0.84-8.03) even with adjustment for the effect of the eHealth team. Thus, reaching a relatively high personal level of physical activity seems more important than aiming for the absolute highest level of physical activity of a group. Addition of more elements to the models (models 6 and 7) attenuated the effects of engagement. The frequency of accelerometer wear (compliance) affected neither BMI nor waist circumference significantly (model 4). The value 0.53 for the standard deviation of the random effect of eHealth team in model 3 shows a maximum difference of 2 kg/m^2^ between teams in reduction of BMI (1.96x2x0.53 kg/m^2^) and the value 1.79 shows a maximum difference of 7 cm in reduction of waist circumference (1.96x2x1.79 cm). Although this exceeds the average reduction in BMI (1.87 kg/m^2^) and in waist circumference (5.6 cm), the variance between participants of eHealth teams hardly changes by adding eHealth team to the model, implying no effect of eHealth team on the reduction in BMI and waist circumference.

**Table 4:** Determinants of reductions in body mass index, at 10 and 23 weeks versus baseline, combined in models using linear mixed models.

Characteristics of program usage: models			∆ BMI^a,b^, kg/m^2^			
			Sources of variance, SD (95% CI)			Β (95% CI)
Model: description	Independent variables (additional)	Categories	Between teams	Between participants		
Model 1: raw			N/A^c^	1.38 (0.97 to 1.96)	N/A	
Model 2: baseline characteristics			N/A	N/A		
	Age				−0.001 (−0.06 to 0.06)	
	Sex	Women			Reference	
		Men			−0.28 (−1.61 to 1.04)	
	BMI at baseline				0.11 (0.01 to 0.21)^h^	
Model 3: model 2 + eHealth team			0.53 (0.10 to 2.73)	1.36 (0.95 to 1.94)		
Model 4: model 3 + compliance			0.39 (0.001 to 0.59)	1.08 (0.65 to 1.78)		
	Compliance^d^	<85%			Reference	
		85%-95%			-0.14 (-1.41 to 1.13)	
		≥95%			0.60 (-0.57 to 1.77)	
Model 5: model 3 + engagement			0.43 (0.001 to 0.60)	1.26 (0.86 to 1.84)		
	Engagement^e^	≤3			Reference	
		4 or 5			1.23 (0.17 to 2.29)	
Model 6: model 3 + MVPA^f^			0.44 (0.001 to 0.60)	1.06 (0.65 to 1.74)		
	MVPA				0.16 (−0.39 to 0.70)	
Model 7: model 3 + compliance + engagement + MVPA			0.50 (−0.00 to 0.64)	1.04 (0.62 to 1.75)		
	Compliance	<85%			Reference	
		85%-95%			−0.09 (−1.37 to 1.19)	
		≥95%			0.13 (−1.22 to 1.47)	
	Engagement	≤3			Reference	
		4 or 5			1.01 (−0.13 to 2.15)	
	MVPA				−0.05 (−0.64 to 0.55)	
	Time	0-10 weeks			Reference	
		0-23 weeks			0.99 (0.35 to 1.62)^g^	

^a^BMI: body mass index.

^b^∆ of outcome = reduction in outcome calculated by measurement at baseline minus measurement at follow-up.

^c^N/A: not applicable.

^d^Compliance is expressed as percentage of days with >10 hours of physical activity registration (accelerometer wear).

^e^Engagement is expressed as the number of times at least 100% of the target was reached (1-5).

^f^MVPA: moderate to vigorous physical activity.

^g^Statistically significant at *P*<.05.

## Discussion

### Principal Findings

In this clinical pilot study in an overweight or obese working population, we evaluated the levels of physical activity during a Web-based gaming intervention using a triaxial accelerometer and we assessed changes in BMI and waist circumference versus baseline. In addition, we evaluated individual characteristics and characteristics of program usage as determinants of our outcomes. We found that levels of physical activity remained high during our intervention and, in addition, reductions in BMI and waist circumference were achieved. Key components for success were social interaction by eHealth teams and the level of engagement. These results indicate that broader implementation of a Web-based gaming intervention with focus on eHealth teams and engagement will be beneficial for overweight and obese individuals, and long-term effects should be studied.

Accelerometer measurements showed a mean MVPA of 86 minutes per day at moderate or vigorous level in our participants, which was high in comparison with an average of 35.5 minutes of MVPA per day in men and 32 minutes in women reported by Hallal et al [33]. The authors reviewed studies with the same wear time criteria of at least 10 hours/day, but subjects were healthy instead of overweight or obese and were observed for a short period of time instead of involved in an active intervention. Our mean MVPA is also high considering that adherence to physical activity guidelines among obese individuals is reported to be even poorer than among healthy weight individuals [5]. When we compare our results with the general Dutch population, we note that a relatively large proportion of our participants met the recommendation of MPA (100% vs 65%) but relatively few met the recommendation of VPA (10% vs 20%). This lack of sufficient vigorous activity was also reported in a systematic review on active video gaming, showing physical activity hardly exceeding 3 METs [34]. The international recommendation to promote and maintain health recommends any person to be moderately active for at least 30 minutes at least 5 days per week, or vigorously active for at least 20 minutes at least 3 days per week, but advises more physical activity for more health benefits [35,36]. The required time and energy expenditure for weight loss is still unclear [37]. MPA, such as walking, is a common, accessible, and inexpensive form of physical activity, which has shown multiple health effects including reduction in BMI [38]. Nevertheless, physical activity at vigorous level is advised for additional health benefits in the WHO (World Health Organization) Global Recommendations on Physical Activity for Health [36], which also requires less time. Therefore, we could debate whether innovative approaches toward Web-based games should also be aimed at increasing the percentage of VPA for further improvement of weight parameters.

Our finding that more accelerometer wear was not associated with more MVPA in multivariable models supports the finding of previous research that just wearing an accelerometer is not sufficient to promote more MVPA [39,40]. There are more benefits of accelerometer wear than just behavioral, though, which extend to objective measurement and providing personalized feedback based on measurements. Objective measurements by body-worn monitors are preferred over self-reported physical activity [41,42], because self-reported information on physical activity is known to be overestimated compared with the actual amount [43], with an even greater inconsistency between self-reported physical activity and that measured using accelerometers among obese individuals [44]. On the other hand, data by accelerometer wear only provide information on the time the device was worn, in contrast to self-reported information, which gives a more general idea of physical activity. Average of 89% days with valid wear time was high in comparison with 73% in another study among workers [18], which could be due to our selection criteria of overweight or obese and highly motivated participants. Other challenges to the use of accelerometers include the loss of the device and incorrect placement of the device [45]. Nevertheless, we will keep considering usage of an accelerometer as a key element of a Web-based gaming intervention for the purpose of accurate registration needed in individual target setting and in competition with others.

Our finding of a mean reduction of 1.87 kg/m^2^ in BMI during the program is high, considering the reported effectiveness of exercise programs among adults who are overweight or obese with a pooled reduction between 0.3 and 0.7 kg/m^2^ [46]. Our results are within the range 0.6 to 4 kg/m^2^ that they reported when a diet was added. BMI was reduced by more than 5%, which means a reduction of obesity-related health risks [47] and a potential gain of psychosocial benefits, such as a decrease in stress and depression [48] and less sick leave at work [49]. Because primarily Web-based interventions are likely to be more cost-effective and have a wider reach, our intervention may be interesting for policy makers and health professionals.

Two important gaming components in our Web-based program were eHealth teams, as a measure of social bonding, and individual engagement, by way of target setting including virtual rewards. Although eHealth team was not associated with the reduction in BMI or waist circumference, both elements showed beneficial effects on the level of physical activity. This is in line with other studies that reported social support to be associated with obesity-specific health-related quality of life [50], with positive health behavior, such us more physical activity and fruit and vegetable intake [51], and with adherence to treatment [52,53]. Kreps and Neuhauser [54] describe how using eHealth for social bonding can really make a difference in enhancing the quality of health care and health promotion effects. A recent study by Zuckerman and Gal-Oz [40] reported no differences in physical activity by adding gaming elements to daily physical activity registration with feedback on progress. The contradiction between these findings and our study could be explained by their short follow-up of several days and thus the novelty effect. A systematic review by Maher et al [55] on the effectiveness of online social networks on changes in diet and weight or physical activity found evidence that online social networks may be effective in changing health behavior. They noted that integrating social networks in gamification is promising and that the user interface of online social networks should be selected carefully so that it is accessible, interactive, contextually tailored, and can be delivered to larger audiences. Online social interaction during our intervention took place on the Web-based forum of the game within and between teams. We suspect an underestimation of the number of Web-based contacts because several teams also communicated through other social media forums. Unclear is why certain teams ended up choosing other social media than that provided by the game, but they may have foreseen that our website would not be accessible anymore after the 23rd week of the game. Although social influence by eHealth teams seems a strong component, the differences between teams were large, and more research is needed to find out how and by whom social support should be delivered and to predict for whom this could work.

Personal targets were set by the individual and reaching targets appeared to be an important gaming component, which can be explained by comparing it to the theory of *flow*, which is popular among video game designers and was described by Eysenbach as one of the popular gamification tactics [12,56]. By setting targets, people become absorbed and engaged in an activity when they are doing something where their skill level is perfectly matched to the challenge level [12]. Nevertheless, maintaining high levels of activity seems challenging because high attrition rates are commonly seen and considered a disadvantage of eHealth [8]. In our game, the average MVPA did not drop despite the common decrease in usage. We suspect that the embedding of target setting in eHealth teams enhanced sustainability of the level of MVPA. Thus, the two gaming components in our Web-based program, that is, eHealth teams and individual engagement, seem to have positively influenced each other.

Blending components of gamification with face-to-face elements may have attributed to our results, although the study design did not allow quantification. A commonly identified benefit of Web-based interventions is their ability to reach a broad population, but Xu et al [57] showed that interventions successful for groups may not always translate to successful behavior change at the individual level. Offering additional face-to-face coaching to individuals with readiness to change behavior may increase intrinsic behavior for personal lifestyle changes by addressing intrinsic motivation [28], thus aiming for behavior changes to be sustained beyond the gaming period.

### Limitations

First, this clinical pilot study was performed among a small number of individuals without preintervention measurements of physical activity and without a control group, leading to a lack of power in some analyses and to the inability to accurately assess the strength of the effects of multiple blended elements [58]. Nevertheless, this compact setting and the increase in physical activity in comparison with self-reported baseline physical activity provided enough information to suggest broader implementation along with a follow-up study including more individuals in a randomized controlled setting.

Second, gaming elements in our intervention were mainly focused on physical activity. Dietary behavior was only addressed during the non-eHealth sessions. Because the focus was on physical activity, the effects on body composition might have been greater than on only BMI and waist circumference, which is beneficial in reducing cardiovascular risk [59]. Although there is sufficient evidence that physical activity in the absence of a dietary intervention can produce weight loss [37,60], these effects could be increased by including healthy diet in the game [29,46]. The mode of delivery should be carefully chosen because the effects differ among technologies and features [10], and the effect on diet should be measured by a food frequency questionnaire.

Finally, the follow-up time of half a year is insufficient to determine the effectiveness of weight loss maintenance and to ignore potential seasonal variations [61].

### Strengths

This study is unique in combining strong and proven effective elements of eHealth with a personalized non-eHealth approach while keeping it fun to engage in. This blended approach is in line with the US guidelines for primary care physicians, advising an initial evaluation by a physician before entering a lifestyle program to increase the chances of long-term success [62,63], and was also advised by Hutchesson et al [10].

The second strength is that we are targeting a high-risk population (selective prevention). Slootmaker et al [18] showed that eHealth interventions are not suitable for all individuals and should be aimed at individuals with risk factors.

Finally, our Web-based program aims at developing, adopting, and maintaining a healthy lifestyle. When proven effective, the prototype can be easily adapted to other target groups such as obese adolescents [64] and children [15], the elderly [20,65], and those in oncology rehabilitation [66].

### Conclusions

This blended Web-based gaming intervention was beneficial in helping participants become physically active above the general recommendation of 30 minutes 5 days per week during the entire intervention period, and a favorable effect on BMI and waist circumference was seen. Promising components in the intervention are teams effects and engagement with the game. Game development should focus on strengthening these elements while keeping the fun factor. Broader implementation and long-term follow-up can provide insights into the sustainable effects on physical activity and weight loss and into who benefits the most from this approach.

## Appendix

Multimedia Appendix 1 CONSORT checklist.

Multimedia Appendix 2 Screenshots of the movement-game.

Multimedia Appendix 3 Univariable association between baseline characteristics and program-usage, and reductions in body mass index and waist circumference (n=51), at 10 and 23 weeks versus baseline, using a linear mixed model.

Multimedia Appendix 4 Determinants of reductions in waist circumference, at 10 and 23 weeks versus baseline, combined in models using linear mixed models.

## Abbreviations

BMI: body mass index
CONSORT-EHEALTH: Consolidated Standards of Reporting Trials of Electronic and Mobile Health Applications and Online Telehealth
MET: metabolic equivalent task
MPA: moderate physical activity
MVPA: moderate to vigorous physical activity
VPA: vigorous physical activity

## References

[1] (2016). World Health Organization.

[2] Lehnert T, Sonntag D, Konnopka A, Riedel-Heller S, König H (2013). Economic costs of overweight and obesity. Best Pract Res Clin Endocrinol Metab.

[3] Ng M, Fleming T, Robinson M, Thomson B, Graetz N, Margono C, Mullany EC, Biryukov S, Abbafati C, Abera SF, Abraham JP, Abu-Rmeileh NM, Achoki T, AlBuhairan FS, Alemu ZA, Alfonso R, Ali MK, Ali R, Guzman NA, Ammar W, Anwari P, Banerjee A, Barquera S, Basu S, Bennett DA, Bhutta Z, Blore J, Cabral N, Nonato IC, Chang J, Chowdhury R, Courville KJ, Criqui MH, Cundiff DK, Dabhadkar KC, Dandona L, Davis A, Dayama A, Dharmaratne SD, Ding EL, Durrani AM, Esteghamati A, Farzadfar F, Fay DF, Feigin VL, Flaxman A, Forouzanfar MH, Goto A, Green MA, Gupta R, Hafezi-Nejad N, Hankey GJ, Harewood HC, Havmoeller R, Hay S, Hernandez L, Husseini A, Idrisov BT, Ikeda N, Islami F, Jahangir E, Jassal SK, Jee SH, Jeffreys M, Jonas JB, Kabagambe EK, Khalifa SE, Kengne AP, Khader YS, Khang Y, Kim D, Kimokoti RW, Kinge JM, Kokubo Y, Kosen S, Kwan G, Lai T, Leinsalu M, Li Y, Liang X, Liu S, Logroscino G, Lotufo PA, Lu Y, Ma J, Mainoo NK, Mensah GA, Merriman TR, Mokdad AH, Moschandreas J, Naghavi M, Naheed A, Nand D, Narayan KM, Nelson EL, Neuhouser ML, Nisar MI, Ohkubo T, Oti SO, Pedroza A, Prabhakaran D, Roy N, Sampson U, Seo H, Sepanlou SG, Shibuya K, Shiri R, Shiue I, Singh GM, Singh JA, Skirbekk V, Stapelberg NJ, Sturua L, Sykes BL, Tobias M, Tran BX, Trasande L, Toyoshima H, van de Vijver S, Vasankari TJ, Veerman JL, Velasquez-Melendez G, Vlassov VV, Vollset SE, Vos T, Wang C, Wang X, Weiderpass E, Werdecker A, Wright JL, Yang YC, Yatsuya H, Yoon J, Yoon S, Zhao Y, Zhou M, Zhu S, Lopez AD, Murray CJ, Gakidou E (2014). Global, regional, and national prevalence of overweight and obesity in children and adults during 1980-2013: a systematic analysis for the Global Burden of Disease Study 2013. Lancet.

[4] Carlsson AC, Ärnlöv J, Sundström J, Michaëlsson K, Byberg L, Lind L (2016). Physical activity, obesity and risk of cardiovascular disease in middle-aged men during a median of 30 years of follow-up. Eur J Prev Cardiol.

[5] Health and Social Care Information Centre, Lifestyle Statistics.

[6] Hildebrandt V, Bernaards C, Hofstetter H (2015). Kenniscentrumsport.

[7] Neve M, Morgan PJ, Jones PR, Collins CE (2010). Effectiveness of web-based interventions in achieving weight loss and weight loss maintenance in overweight and obese adults: a systematic review with meta-analysis. Obes Rev.

[8] Eysenbach G (2005). The law of attrition. J Med Internet Res.

[9] Mouthaan J, Sijbrandij M, de Vries G, Reitsma JB, van de Schoot R, Goslings JC, Luitse JS, Bakker FC, Gersons BP, Olff M (2013). Internet-based early intervention to prevent posttraumatic stress disorder in injury patients: randomized controlled trial. J Med Internet Res.

[10] Hutchesson MJ, Rollo ME, Krukowski R, Ells L, Harvey J, Morgan PJ, Callister R, Plotnikoff R, Collins CE (2015). eHealth interventions for the prevention and treatment of overweight and obesity in adults: a systematic review with meta-analysis. Obes Rev.

[11] Poirier J, Bennett WL, Jerome GJ, Shah NG, Lazo M, Yeh H, Clark JM, Cobb NK (2016). Effectiveness of an activity tracker- and internet-based adaptive walking program for adults: a randomized controlled trial. J Med Internet Res.

[12] Cugelman B (2013). Gamification: what it is and why it matters to digital health behavior change developers. JMIR Serious Games.

[13] Sawada SS (2014). Physical fitness for health. J Phys Fitness Sports Med.

[14] Mora S, Cook N, Buring JE, Ridker PM, Lee I (2007). Physical activity and reduced risk of cardiovascular events: potential mediating mechanisms. Circulation.

[15] Maddison R, Foley L, Ni MC, Jiang Y, Jull A, Prapavessis H, Hohepa M, Rodgers A (2011). Effects of active video games on body composition: a randomized controlled trial. Am J Clin Nutr.

[16] Dachs R (2007). Exercise is an effective intervention in overweight and obese patients. Am Fam Physician.

[17] Wadden TA, Webb VL, Moran CH, Bailer BA (2012). Lifestyle modification for obesity: new developments in diet, physical activity, and behavior therapy. Circulation.

[18] Slootmaker SM, Chinapaw MJ, Schuit AJ, Seidell JC, Van Mechelen W (2009). Feasibility and effectiveness of online physical activity advice based on a personal activity monitor: randomized controlled trial. J Med Internet Res.

[19] Bravata DM, Smith-Spangler C, Sundaram V, Gienger AL, Lin N, Lewis R, Stave CD, Olkin I, Sirard JR (2007). Using pedometers to increase physical activity and improve health: a systematic review. JAMA.

[20] Wijsman CA, Westendorp RG, Verhagen EA, Catt M, Slagboom PE, de Craen AJ, Broekhuizen K, van Mechelen W, van Heemst D, van der Ouderaa F, Mooijaart SP (2013). Effects of a web-based intervention on physical activity and metabolism in older adults: randomized controlled trial. J Med Internet Res.

[21] Staiano AE, Abraham AA, Calvert SL (2012). The Wii club: gaming for weight loss in overweight and obese youth. Games Health J.

[22] Allam A, Kostova Z, Nakamoto K, Schulz PJ (2015). The effect of social support features and gamification on a Web-based intervention for rheumatoid arthritis patients: randomized controlled trial. J Med Internet Res.

[23] Lustria ML, Cortese J, Noar SM, Glueckauf RL (2009). Computer-tailored health interventions delivered over the Web: review and analysis of key components. Patient Educ Couns.

[24] Reid RD, Morrin LI, Higginson LA, Wielgosz A, Blanchard C, Beaton LJ, Nelson C, McDonnell L, Oldridge N, Wells GA, Pipe AL (2012). Motivational counselling for physical activity in patients with coronary artery disease not participating in cardiac rehabilitation. Eur J Prev Cardiol.

[25] Simpson SA, McNamara R, Shaw C, Kelson M, Moriarty Y, Randell E, Cohen D, Alam MF, Copeland L, Duncan D, Espinasse A, Gillespie D, Hill A, Owen-Jones E, Tapper K, Townson J, Williams S, Hood K (2015). A feasibility randomised controlled trial of a motivational interviewing-based intervention for weight loss maintenance in adults. Health Technol Assess.

[26] Hardcastle SJ, Taylor AH, Bailey MP, Harley RA, Hagger MS (2013). Effectiveness of a motivational interviewing intervention on weight loss, physical activity and cardiovascular disease risk factors: a randomised controlled trial with a 12-month post-intervention follow-up. Int J Behav Nutr Phys Act.

[27] Eysenbach G, CONSORT-EHEALTH Group (2011). CONSORT-EHEALTH: improving and standardizing evaluation reports of web-based and mobile health interventions. J Med Internet Res.

[28] Miller WR, Rollnick S (2012). Motivational Interviewing: Helping People Change.

[29] (2008). Kwaliteitsinstituut voor de Gezondheidszorg CBO.

[30] Kemper H, Ooijendijk W, Stiggelbout M (2000). Consensus over de Nederlandse norm voor gezond bewegen (consensus on the Dutch standard for healthy physical activity). Tijdschrift voor gezondheidswetenschappen.

[31] Activ8.

[32] Nederlands Huisartsen Genootschap.

[33] Hallal PC, Andersen LB, Bull FC, Guthold R, Haskell W, Ekelund U, Lancet Physical Activity Series Working Group (2012). Global physical activity levels: surveillance progress, pitfalls, and prospects. Lancet.

[34] Peng W, Crouse JC, Lin J (2013). Using active video games for physical activity promotion: a systematic review of the current state of research. Health Educ Behav.

[35] (2013). Centraal Bureau voor de Statistiek (CBS), Rijksinstituut voor Volksgezondheid en Milieu (RIVM), and Gemeentelijke Gezondheidsdiensten (GGD-en).

[36] (2010). World Health Organization.

[37] Strasser B (2013). Physical activity in obesity and metabolic syndrome. Ann N Y Acad Sci.

[38] Hanson S, Jones A (2015). Is there evidence that walking groups have health benefits? a systematic review and meta-analysis. Br J Sports Med.

[39] Ho V, Simmons RK, Ridgway CL, van Sluijs EM, Bamber DJ, Goodyer IM, Dunn VJ, Ekelund U, Corder K (2013). Is wearing a pedometer associated with higher physical activity among adolescents?. Prev Med.

[40] Zuckerman O, Gal-Oz A (2014). Deconstructing gamification: evaluating the effectiveness of continuous measurement, virtual rewards, and social comparison for promoting physical activity. Pers Ubiquit Comput.

[41] Godfrey A, Rochester L (2015). Body-worn monitors: a lot done, more to do. J Epidemiol Community Health.

[42] Compernolle S, Vandelanotte C, Cardon G, De Bourdeaudhuij I, De Cocker K (2015). Effectiveness of a web-based, computer-tailored, pedometer-based physical activity intervention for adults: a cluster randomized controlled trial. J Med Internet Res.

[43] Dyrstad SM, Hansen BH, Holme IM, Anderssen SA (2014). Comparison of self-reported versus accelerometer-measured physical activity. Med Sci Sports Exerc.

[44] Warner ET, Wolin KY, Duncan DT, Heil DP, Askew S, Bennett GG (2012). Differential accuracy of physical activity self-report by body mass index. Am J Health Behav.

[45] Sharpe PA, Wilcox S, Rooney LJ, Strong D, Hopkins-Campbell R, Butel J, Ainsworth B, Parra-Medina D (2011). Adherence to accelerometer protocols among women from economically disadvantaged neighborhoods. J Phys Act Health.

[46] Shaw K, Gennat H, O'Rourke P, Del Mar C (2006). Exercise for overweight or obesity. Cochrane Database Syst Rev.

[47] Jensen MD, Ryan DH (2014). New obesity guidelines: promise and potential. JAMA.

[48] Elder CR, Gullion CM, Funk KL, Debar LL, Lindberg NM, Stevens VJ (2012). Impact of sleep, screen time, depression and stress on weight change in the intensive weight loss phase of the LIFE study. Int J Obes (Lond).

[49] Neovius K, Johansson K, Kark M, Neovius M (2009). Obesity status and sick leave: a systematic review. Obes Rev.

[50] Herzer M, Zeller MH, Rausch JR, Modi AC (2011). Perceived social support and its association with obesity-specific health-related quality of life. J Dev Behav Pediatr.

[51] Emmons KM, Barbeau EM, Gutheil C, Stryker JE, Stoddard AM (2007). Social influences, social context, and health behaviors among working-class, multi-ethnic adults. Health Educ Behav.

[52] DiMatteo MR (2004). Social support and patient adherence to medical treatment: a meta-analysis. Health Psychol.

[53] Baum A, Revenson T, Singer J (2012). Handbook of health psychology, second edition.

[54] Kreps GL, Neuhauser L (2010). New directions in eHealth communication: opportunities and challenges. Patient Educ Couns.

[55] Maher CA, Lewis LK, Ferrar K, Marshall S, De Bourdeaudhuij I, Vandelanotte C (2014). Are health behavior change interventions that use online social networks effective? a systematic review. J Med Internet Res.

[56] Csikszentmihalyi M (2008). Flow: The Psychology of Optimal Experience.

[57] Xu Y, Poole ES, Miller A (2012). This is not a one-horse race: understanding player types in multiplayer pervasive health games for youth.

[58] Danaher BG, Seeley JR (2009). Methodological issues in research on web-based behavioral interventions. Ann Behav Med.

[59] Stoner L, Rowlands D, Morrison A, Credeur D, Hamlin M, Gaffney K, Lambrick D, Matheson A (2016). Efficacy of exercise intervention for weight loss in overweight and obese adolescents: meta-analysis and implications. Sports Med.

[60] Slentz CA, Duscha BD, Johnson JL, Ketchum K, Aiken LB, Samsa GP, Houmard JA, Bales CW, Kraus WE (2004). Effects of the amount of exercise on body weight, body composition, and measures of central obesity: STRRIDE--a randomized controlled study. Arch Intern Med.

[61] Rich C, Griffiths LJ, Dezateux C (2012). Seasonal variation in accelerometer-determined sedentary behaviour and physical activity in children: a review. Int J Behav Nutr Phys Act.

[62] Ryan D, Heaner M (2014). Guidelines (2013) for managing overweight and obesity in adults. Preface to the full report. Obesity (Silver Spring).

[63] Kushner RF, Ryan DH (2014). Assessment and lifestyle management of patients with obesity: clinical recommendations from systematic reviews. JAMA.

[64] Chen J, Wilkosz ME (2014). Efficacy of technology-based interventions for obesity prevention in adolescents: a systematic review. Adolesc Health Med Ther.

[65] Skjæret N, Nawaz A, Morat T, Schoene D, Helbostad JL, Vereijken B (2016). Exercise and rehabilitation delivered through exergames in older adults: An integrative review of technologies, safety and efficacy. Int J Med Inform.

[66] Zimmer P, Baumann FT, Oberste Max, Wright P, Garthe A, Schenk A, Elter T, Galvao DA, Bloch W, Hübner ST, Wolf F (2016). Effects of exercise interventions and physical activity behavior on cancer related cognitive impairments: a systematic review. Biomed Res Int.

