# Supplementary material for: A Blended Web-Based Gaming Intervention on Changes in Physical Activity for Overweight and Obese Employees: Influence and Usage in an Experimental Pilot Study
Source: JMIR Serious Games. 2017 Apr 3;5(2):e6. doi: 10.2196/games.6421 (PMC5394263; doi:10.2196/games.6421)
Supplement: Multimedia Appendix 1 [file games_v5i2e6_app1.pdf]

Your name *

First Last

Kouwenhoven-Pasmooij

Primary Affiliation (short), City, Country *

University of Toronto, Toronto, Canada

Erasmus MC, University

Your e-mail address *

abc@google.com

t.kouwenhoven@erasmu

Do you want to be acknowledged (with your name and affilitation) in the CONSORT-EHEALTH publication? *

(provided you make contributions below. Please refer to the last question if you also wish to be a co-author of the Elaboration manuscript)

- ○ yes
- ◉ no

Your role/experience with ehealth-trials

Which of the following describes you (multiple may apply)

- ☐ I have experience with conducting ehealth studies myself, but no RCTs
- ☑ I have conducted ehealth RCTs
- ☑ I have read many ehealth RCT/evaluation reports
- ☐ I have experience mainly from a consumer/patient point of view
- ☐ I have experience mainly from a policy/implementation/decision-maker point of view

Other guidelines

Are you aware of any other guidelines that should be cited? (give references and provide a short description)

TITLE AND ABSTRACT

1a) Identification as a randomized trial in the title

1a-i) Identify the mode of delivery in the title

Identify the mode of delivery. Preferably use “web-based” and/or “mobile” and/or “electronic game” in the title. Avoid ambiguous terms like “online”, “virtual”, “interactive”. Use “Internet-based” only if Intervention includes non-web-based Internet components (e.g. email), use

“computer-based” or “electronic” only if offline products are used. Use “virtual” only in the context of “virtual reality” (3-D worlds). Use “online” only in the context of “online support groups”. Complement or substitute product names with broader terms for the class of products (such as “mobile” or “smart phone” instead of “iphone”), especially if the application runs on different platforms.

	1	2	3	4	5	
subitem not at all important	◯	◯	◯	◯	◯	essential

Comment on subitem 1a-i)

It is a web-based intervention.

1a-ii) Non-web-based components or important co-interventions in title

Mention non-web-based components or important co-interventions in title, if any (e.g., “with telephone support”).

	1	2	3	4	5	
subitem not at all important	◯	◯	◯	◯	◯	essential

Comment on subitem 1a-ii)

"blended" is mentioned in the title.

1a-iii) Primary condition or target group in the title

Mention primary condition or target group in the title, if any (e.g., “for children with Type I Diabetes”) Example: A Web-based and Mobile Intervention with Telephone Support for Children with Type I Diabetes: Randomized Controlled Trial

	1	2	3	4	5	
subitem not at all important	◯	◯	◯	◯	◯	essential

Comment on subitem 1a-iii)

overweight and obese employees are our target group.

Add a subitem under CONSORT item 1a

1b) Structured summary of trial design, methods, results, and conclusions

NPT extension: Description of experimental treatment, comparator, care providers, centers, and blinding status.

1b-i) Key features/functionalities/components of the intervention and comparator in the abstract

Mention key features/functionalities/components of the intervention and comparator in the abstract. If possible, also mention theories and principles used for designing the site. Keep in mind the needs of systematic reviewers and indexers by including important synonyms.

	1	2	3	4	5	
subitem not at all important	○	○	○	○	○	essential

Comment on subitem 1b-i)

overweight and obese employees recieved a blended web-based gaming intervention, which was directed at increasing PA and reducing body mass index (BMI) and waist circumference

1b-ii) Level of human involvement in the abstract

Clarify the level of human involvement in the abstract, e.g., use phrases like “fully automated” vs. “therapist/nurse/care provider/physician-assisted” (mention number and expertise of providers involved, if any).

	1	2	3	4	5	
subitem not at all important	○	○	○	○	○	essential

Comment on subitem 1b-ii)

the program is administered in a blended care approach, meaning that eHealth-elements were blended with face-to-face contacts. These non-eHealth components were an individual session with an occupational health physician by the method of motivational interviewing and five multidisciplinary group-sessions.

1b-iii) Open vs. closed, web-based (self-assessment) vs. face-to-face assessments in abstract

Open vs. closed, web-based (self-assessment) vs. face-to-face assessments in abstract: Mention how participants were recruited (online vs. offline), e.g., from an open access website (open trial) or from a clinic or other closed user group (closed trial), and clarify if this was a purely web-based trial, or there were face-to-face components (as part of the intervention or for assessment). Clearly say if outcomes were self-assessed through questionnaires (as common in web-based trials).

	1	2	3	4	5	
subitem not at all important	○	○	○	○	○	essential

Comment on subitem 1b-iii)

Participants were recruited via the company's web. "Primary outcome was PA, defined as amount of time at MET≥3, as measured by an accelerometer during the game. Secondary outcomes were reductions in BMI and waist circumference, measured at baseline, and 10 and 23 weeks after the start of the program. "

1b-iv) Results in abstract must contain use data

Report number of participants enrolled/assessed in each group, the use/uptake of the intervention (e.g., attrition/adherence metrics, use over time, number of logins etc.), in addition to primary/secondary outcomes.

	1	2	3	4	5	
subitem not at all important	○	○	○	○	○	essential

Comment on subitem 1b-iv)

we observed 52 health-care employees. The mean age of participants was 48.1 years; the majority of participants were female (82%). The mean PA was 86 minutes per day, ranging from 6.5 to 223 minutes, which remained fairly constant during the game. Mean BMI was reduced by 1.87 kg/m2 (5.7%) and waist circumference by 5.8 cm (4.8%). Results of the univariable model showed that compliance, engagement and eHealth-teams were associated with more PA, which remained [illegible]

1b-v) Conclusions/Discussions in abstract for negative trials

Conclusions/Discussions in abstract for negative trials: Discuss the primary outcome - if the trial is negative (primary outcome not changed), and the intervention was not used, discuss whether negative results are attributable to lack of uptake and discuss reasons.

	1	2	3	4	5	
subitem not at all important	○	○	○	○	○	essential

Comment on subitem 1b-v)

This blended web-based gaming intervention was beneficial for overweight workers in being physically active above the recommended activity levels during the entire intervention-period, and a beneficial influence on BMI and waist circumference was seen. Promising components in the intervention are eHealth-teams and engagement with the game. Broader implementation and long-term follow-up can provide insights into the remaining effects on PA and weight [illegible]

Add a subitem under CONSORT item 1b

INTRODUCTION

2a) Scientific background and explanation of rationale

2a-i) Problem and the type of system/solution

Describe the problem and the type of system/solution that is object of the study: intended as stand-alone intervention vs. incorporated in broader health care program? [1] Intended for a particular patient population? [1] Goals of the intervention, e.g., being more cost-effective to other interventions [1], replace or complement other solutions? (Note: Details about the intervention are provided in “Methods” under 5)

	1	2	3	4	5	
subitem not at all important	○	○	○	○	○	essential

Comment on subitem 2a-i)

" Adherence to PA recommendations among obese individuals is poor"

"Aiming for a both effective and efficient intervention with the blended usage of eHealth components and non-eHealth, we constructed our program and implemented it in a pilot setting. The results of this pilot will inform us whether broader implementation with longer follow-up is useful for this target [illegible]

2a-ii) Scientific background, rationale

Scientific background, rationale: What is known about the (type of) system that is the object of the study (be sure to discuss the use of similar systems for other conditions/diagnoses, if appropiate), motivation for the study, i.e. what are the reasons for and what is the context for this specific study, from which stakeholder viewpoint is the study performed, potential impact of findings [2]. Briefly justify the choice of the comparator.

	1	2	3	4	5	
subitem not at all important	○	○	○	○	○	essential

Comment on subitem 2a-ii)

Scientific background and rationale are clearly stated in the introduction.

Add a subitem under CONSORT item 2a

2b) Specific objectives or hypotheses

(note: Contrary to STARE-HI we do not recommend to mention IRB approval in this section - JMIR and other journals typically recommend this as a subheading under “methods”. CONSORT-EHEALTH has a separate item for ethical considerations)

(no EHEALTH-specific subitems under CONSORT item 2b)

Comment below to suggest a subitem

Therefore, the aims of this study were to analyze the sustainability of PA during the game, and to assess changes in body mass index (BMI) and waist circumference. In addition, we aimed to assess the influence of compliance, engagement and eHealth-teams on these outcomes.

METHODS

3a) Description of trial design (such as parallel, factorial) including allocation ratio

(no EHEALTH-specific subitems under CONSORT item 3a)

Comment below to suggest a subitem

This pilot study evaluates a blended web-based gaming intervention for overweight and obese employees to become more physically active and adopt a healthy diet in a way that suits their personal preferences and abilities, and ultimately, to lose weight.
The program consists of a face-to-face intake-session with the occupational health physician (OP), five group-sessions, and a 20-week movement-game, which is played real-life using [illegible]

3b) Important changes to methods after trial commencement (such as eligibility criteria), with reasons

3b-i) Bug fixes, Downtimes, Content Changes

Bug fixes, Downtimes, Content Changes: ehealth systems are often dynamic systems. A description of changes to methods therefore also includes important changes made on the intervention or comparator during the trial (e.g., major bug fixes or changes in the functionality or content) (5-iii) and other "unexpected events" that may have influenced study design such as staff changes, system failures/downtimes, etc. [2].

	1	2	3	4	5	
subitem not at all important	○	○	○	○	○	essential

Comment on subitem 3b-i)

During the program, no interfering bug fixing was needed.

Add a subitem under CONSORT item 3b

4a) Eligibility criteria for participants

4a-i) Computer / Internet literacy

Computer / Internet literacy is often an implicit “de facto” eligibility criterion - this should be explicitly clarified [1].

	1	2	3	4	5	
subitem not at all important	○	○	○	○	○	essential

Comment on subitem 4a-i)

Because of the web-based approach, affinity with computers was desirable, but only computer-accessibility was required.

4a-ii) Open vs. closed, web-based vs. face-to-face assessments:

Open vs. closed, web-based vs. face-to-face assessments: Mention how participants were recruited (online vs. offline), e.g., from an open access website or from a clinic, and clarify if this was a purely web-based trial, or there were face-to-face components (as part of the intervention or for assessment), i.e., to what degree got the study team to know the participant. In online-only trials, clarify if participants were quasi-anonymous and whether having multiple identities was possible or whether technical or logistical measures (e.g., cookies, email confirmation, phone calls) were used to detect/prevent these.

	1	2	3	4	5	
subitem not at all important	○	○	○	○	○	essential

Comment on subitem 4a-ii)

The way of recruitment, how many participants were recruited, and in of which company is stated in the 'materials and methods'-part of the manuscript.

4a-iii) Information giving durnig recruitment

Information given during recruitment. Specify how participants were briefed for recruitment and in the informed consent procedures (e.g., publish the informed consent documentation as appendix, see also item X26), as this information may have an effect on user self-selection, user expectation and may also bias results.

	1	2	3	4	5	
subitem not at all important	○	○	○	○	○	essential

Comment on subitem 4a-iii)

The information published on the company's web and the patient information were in Dutch, and not included in the manuscript.

Add a subitem under CONSORT item 4a

4b) Settings and locations where the data were collected

4b-i) Report if outcomes were (self-)assessed through online questionnaires

Clearly report if outcomes were (self-)assessed through online questionnaires (as common in web-based trials) or otherwise.

	1	2	3	4	5	
subitem not at all important	○	○	○	○	○	essential

Comment on subitem 4b-i)

Online questionnaires were not used to assess our outcomes.

4b-ii) Report how institutional affiliations are displayed

"Report how institutional affiliations are displayed to potential participants [on ehealth media], as affiliations with prestigious hospitals or universities may affect volunteer rates, use, and reactions with regards to an intervention" [1].

	1	2	3	4	5	
subitem not at all important	○	○	○	○	○	essential

Comment on subitem 4b-ii)

This is not specifically addressed in manuscript: the study was completely performed by the hospital they were employed at.

Add a subitem under CONSORT item 4b

5) The interventions for each group with sufficient details to allow replication, including how and when they were actually administered

5-i) Mention names, credential, affiliations of the developers, sponsors, and owners

Mention names, credential, affiliations of the developers, sponsors, and owners [6] (if authors/evaluators are owners or developer of the software, this needs to be declared in a "Conflict of interest" section).

	1	2	3	4	5	
subitem not at all important	○	○	○	○	○	essential

Comment on subitem 5-i)

"Registration of PA was performed by the Activ8 system, which is a small 3D-accelerometer that is worn in the pocket of any pants or with a leg-strap on the upper leg [31]. The game-coach handed out instructions on installing the Activ8-software to the computer, and assisted if necessary."Participants could actually see the name of the system on the device and also this brand when uploading the device.

5-ii) Describe the history/development process

Describe the history/development process of the application and previous formative evaluations (e.g., focus groups, usability testing), as these will have an impact on adoption/use rates and help with interpreting results.

	1	2	3	4	5	
subitem not at all important	○	○	○	○	○	essential

Comment on subitem 5-ii)

"The main idea was developed and tested in 2010, and upgraded to the current version in 2013, which was tested by a test-group before implementation in our study population"

5-iii) Revisions and updating

Revisions and updating. Clearly mention the date and/or version number of the application/intervention (and comparator, if applicable) evaluated, or describe whether the intervention underwent major changes during the evaluation process, or whether the development and/or content was “frozen” during the trial. Describe dynamic components such as news feeds or changing content which may have an impact on the replicability of the intervention (for unexpected events see item 3b).

	1	2	3	4	5	
subitem not at all important	○	○	○	○	○	essential

Comment on subitem 5-iii)

not applicable, as the intervention was used in full version.

5-iv) Quality assurance methods

Provide information on quality assurance methods to ensure accuracy and quality of information provided [1], if applicable.

	1	2	3	4	5	
subitem not at all important	○	○	○	○	○	essential

Comment on subitem 5-iv)

"Confidentiality of users was ensured by only using first names in the game. To ensure security of content and users, the game used password-protected accounts, encrypted password storage, encrypted login details, and secure external servers. "
The brand of the scale is mentioned, and waist circumference was always measured by the same OP according to guidelines.

5-v) Ensure replicability by publishing the source code, and/or providing screenshots/screen-capture video, and/or providing flowcharts of the algorithms used

Ensure replicability by publishing the source code, and/or providing screenshots/screen-capture video, and/or providing flowcharts of the algorithms used. Replicability (i.e., other researchers should in principle be able to replicate the study) is a hallmark of scientific reporting.

	1	2	3	4	5	
subitem not at all important	○	○	○	○	○	essential

Comment on subitem 5-v)

Screenshots are provided as multimedia appendix.

5-vi) Digital preservation

Digital preservation: Provide the URL of the application, but as the intervention is likely to change or disappear over the course of the years; also make sure the intervention is archived (Internet Archive, webcitation.org, and/or publishing the source code or screenshots/videos alongside the article). As pages behind login screens cannot be archived, consider creating demo pages which are accessible without login.

	1	2	3	4	5	
subitem not at all important	○	○	○	○	○	essential

Comment on subitem 5-vi)

Screenshots are provided as multimedia appendix.

5-vii) Access

Access: Describe how participants accessed the application, in what setting/context, if they had to pay (or were paid) or not, whether they had to be a member of specific group. If known, describe how participants obtained "access to the platform and Internet" [1]. To ensure access

for editors/reviewers/readers, consider to provide a “backdoor” login account or demo mode for reviewers/readers to explore the application (also important for archiving purposes, see vi).

	1	2	3	4	5	
subitem not at all important	◯	◯	◯	◯	◯	essential

Comment on subitem 5-vii)

In the methods section is clearly stated how much was paid by the participants, and how and when the accessed the online platform.

5-viii) Mode of delivery, features/functionalities/components of the intervention and comparator, and the theoretical framework

Describe mode of delivery, features/functionalities/components of the intervention and comparator, and the theoretical framework [6] used to design them (instructional strategy [1], behaviour change techniques, persuasive features, etc., see e.g., [7, 8] for terminology). This includes an in-depth description of the content (including where it is coming from and who developed it) [1],” whether [and how] it is tailored to individual circumstances and allows users to track their progress and receive feedback” [6]. This also includes a description of communication delivery channels and – if computer-mediated communication is a component – whether communication was synchronous or asynchronous [6]. It also includes information on presentation strategies [1], including page design principles, average amount of text on pages, presence of hyperlinks to other resources, etc. [1].

	1	2	3	4	5	
subitem not at all important	◯	◯	◯	◯	◯	essential

Comment on subitem 5-viii)

The mode of delivery is divided in three items: the session with the OP, the group-sessions and the movement-game.

5-ix) Describe use parameters

Describe use parameters (e.g., intended “doses” and optimal timing for use) [1]. Clarify what instructions or recommendations were given to the user, e.g., regarding timing, frequency, heaviness of use [1], if any, or was the intervention used ad libitum.

	1	2	3	4	5	
subitem not at all important	◯	◯	◯	◯	◯	essential

Comment on subitem 5-ix)

"During the game, the Activ8 needed to be worn at all times, except during swimming and sleeping. "

5-x) Clarify the level of human involvement

Clarify the level of human involvement (care providers or health professionals, also technical assistance) in the e-intervention or as co-intervention (detail number and expertise of professionals involved, if any, as well as "type of assistance offered, the timing and frequency of the support, how it is initiated, and the medium by which the assistance is delivered" [6]. It may be necessary to distinguish between the level of human involvement required for the trial, and the level of human involvement required for a routine application outside of a RCT setting (discuss under item 21 – generalizability).

	1	2	3	4	5	
subitem not at all important	○	○	○	○	○	essential

Comment on subitem 5-x)

"the sessions were alternately given by a physician, a dietician, a physical therapist, and a psychologist. "
"An independent 'game-coach' randomly divided the participants "

5-xi) Report any prompts/reminders used

Report any prompts/reminders used: Clarify if there were prompts (letters, emails, phone calls, SMS) to use the application, what triggered them, frequency etc [1]. It may be necessary to distinguish between the level of prompts/reminders required for the trial, and the level of prompts/reminders for a routine application outside of a RCT setting (discuss under item 21 – generalizability).

	1	2	3	4	5	
subitem not at all important	○	○	○	○	○	essential

Comment on subitem 5-xi)

"Every two weeks, an automatic e-mail was sent to the participants, providing general information on multiple lifestyle aspects related to the upcoming continent. If participants failed to log on to the game-website for more than two weeks, an e-mail reminder was sent by the game-coach"

5-xii) Describe any co-interventions (incl. training/support)

Describe any co-interventions (incl. training/support): Clearly state any "interventions that are provided in addition to the targeted eHealth intervention" [1], as ehealth intervention may not be designed as stand-alone intervention. This includes training sessions and support [1]. It may be necessary to distinguish between the level of training required for the trial, and the level of training for a routine application outside of a RCT setting (discuss under item 21 – generalizability.

	1	2	3	4	5	
subitem not at all important	○	○	○	○	○	essential

Comment on subitem 5-xii)

no co-interventions were used

Add a subitem under CONSORT item 5

6a) Completely defined pre-specified primary and secondary outcome measures, including how and when they were assessed

6a-i) Online questionnaires: describe if they were validated for online use [6] and apply CHERRIES items to describe how the questionnaires were designed/deployed

If outcomes were obtained through online questionnaires, describe if they were validated for online use [6] and apply CHERRIES items to describe how the questionnaires were designed/deployed [9].

	1	2	3	4	5	
subitem not at all important	○	○	○	○	○	essential

Comment on subitem 6a-i)

Online questionnaires were not used for this intervention.
Our primary outcome physical activity was measured by a 3D accelerometer, BMI was measured by a calibrated scale, and waist circumference was measured by the same OP at all times.

6a-ii) Describe whether and how "use" (including intensity of use/dosage) was defined/measured/monitored

Describe whether and how "use" (including intensity of use/dosage) was defined/measured/monitored (logins, logfile analysis, etc.). Use/adoption metrics are important process outcomes that should be reported in any ehealth trial.

	1	2	3	4	5	
subitem not at all important	○	○	○	○	○	essential

Comment on subitem 6a-ii)

Use was defined by compliance (the number of days the accelerometer was intensily worn), and engagement (the number of times an individual award was won), amount of logins to the program, and number of messages at the forum.

6a-iii) Describe whether, how, and when qualitative feedback from participants was obtained

Describe whether, how, and when qualitative feedback from participants was obtained (e.g., through emails, feedback forms, interviews, focus groups).

	1	2	3	4	5	
subitem not at all important	○	○	○	○	○	essential

Comment on subitem 6a-iii)

Not applicable for out outcomes. We will use focus groups before implementation at a larger scale.

Add a subitem under CONSORT item 6a

6b) Any changes to trial outcomes after the trial commenced, with reasons

(no EHEALTH-specific subitems under CONSORT item 6b)

Comment below to suggest a subitem

No changes to the trial outcomes.

7a) How sample size was determined

NPT: When applicable, details of whether and how the clustering by care provides or centers was addressed

7a-i) Describe whether and how expected attrition was taken into account when calculating the sample size

Describe whether and how expected attrition was taken into account when calculating the sample size.

	1	2	3	4	5	
subitem not at all important	○	○	○	○	○	essential

Comment on subitem 7a-i)

In this clinical pilot study we analyzed data based on four actual programs, in order to gain information on working elements of our intervention.

Add a subitem under CONSORT item 7a

7b) When applicable, explanation of any interim analyses and stopping guidelines

(no EHEALTH-specific subitems under CONSORT item 7b)

Comment below to suggest a subitem

not applicable

8a) Method used to generate the random allocation sequence

NPT: When applicable, how care providers were allocated to each trial group

(no EHEALTH-specific subitems under CONSORT item 8a)

Comment below to suggest a subitem

"An independent ‘game-coach’ randomly divided the participants of one program into two eHealth-teams,"

8b) Type of randomisation; details of any restriction (such as blocking and block size)

(no EHEALTH-specific subitems under CONSORT item 8b)

Comment below to suggest a subitem

not applicable

9) Mechanism used to implement the random allocation sequence (such as sequentially numbered containers), describing any steps taken to conceal the sequence until interventions were assigned

(no EHEALTH-specific subitems under CONSORT item 9)

Comment below to suggest a subitem

not applicable

10) Who generated the random allocation sequence, who enrolled participants, and who assigned participants to interventions

(no EHEALTH-specific subitems under CONSORT item 10)

Comment below to suggest a subitem

not applicable

11a) If done, who was blinded after assignment to interventions (for example, participants, care providers, those assessing outcomes) and how

NPT: Whether or not administering co-interventions were blinded to group assignment

11a-i) Specify who was blinded, and who wasn't

Specify who was blinded, and who wasn't. Usually, in web-based trials it is not possible to blind the participants [1, 3] (this should be clearly acknowledged), but it may be possible to blind outcome assessors, those doing data analysis or those administering co-interventions (if any).

	1	2	3	4	5	
subitem not at all important	○	○	○	○	○	essential

Comment on subitem 11a-i)

participants and care providers were not blinded.

11a-ii) Discuss e.g., whether participants knew which intervention was the "intervention of interest" and which one was the "comparator"

Informed consent procedures (4a-ii) can create biases and certain expectations - discuss e.g., whether participants knew which intervention was the "intervention of interest" and which one was the "comparator".

	1	2	3	4	5	
subitem not at all important	○	○	○	○	○	essential

Comment on subitem 11a-ii)

not applicable

Add a subitem under CONSORT item 11a

11b) If relevant, description of the similarity of interventions

(this item is usually not relevant for ehealth trials as it refers to similarity of a placebo or sham intervention to a active medication/intervention)

(no EHEALTH-specific subitems under CONSORT item 11b)

Comment below to suggest a subitem

not applicable

12a) Statistical methods used to compare groups for primary and secondary outcomes

NPT: When applicable, details of whether and how the clustering by care providers or centers was addressed

12a-i) Imputation techniques to deal with attrition / missing values

Imputation techniques to deal with attrition / missing values: Not all participants will use the intervention/comparator as intended and attrition is typically high in ehealth trials. Specify how participants who did not use the application or dropped out from the trial were treated in the statistical analysis (a complete case analysis is strongly discouraged, and simple imputation techniques such as LOCF may also be problematic [4]).

	1	2	3	4	5	
subitem not at all important	○	○	○	○	○	essential

Comment on subitem 12a-i)

"We excluded data of one participant because of pregnancy. " "Univariable and multivariable analyses were performed using linear mixed models to account for the within-subject correlations due to intra-team effects and, in the case of BMI and waist circumference, for repeated measurements. "By the multivariable mixed models, any missing independent variables were automatically imputed.

Add a subitem under CONSORT item 12a

12b) Methods for additional analyses, such as subgroup analyses and adjusted analyses

(no EHEALTH-specific subitems under CONSORT item 12b)

Comment below to suggest a subitem

The multivariable mixed models were adjusted for gender, age and education.

X26) (not a CONSORT item)

X26-i) Comment on ethics committee approval

	1	2	3	4	5	
subitem not at all important	○	○	○	○	○	essential

Comment on subitem X26-i)

x26-ii) Outline informed consent procedures

Outline informed consent procedures e.g., if consent was obtained offline or online (how? Checkbox, etc.?), and what information was provided (see 4a-ii). See [6] for some items to be included in informed consent documents.

	1	2	3	4	5	
subitem not at all important	○	○	○	○	○	essential

Comment on subitem X26-ii)

Informed consents were obtained offline. The

X26-iii) Safety and security procedures

Safety and security procedures, incl. privacy considerations, and "any steps taken to reduce the likelihood or detection of harm (e.g., education and training, availability of a hotline)" [1].

	1	2	3	4	5	
subitem not at all important	○	○	○	○	○	essential

Comment on subitem X26-iii)

Add a subitem under item X26

RESULTS

13a) For each group, the numbers of participants who were randomly assigned, received intended treatment, and were analysed for the primary outcome

NPT: The number of care providers or centers performing the intervention in each group and the number of patients treated by each care provider in each center

(no EHEALTH-specific subitems under CONSORT item 13a)

Comment below to suggest a subitem

"In total, 52 employees participated in this program, of whom one was excluded from analyses because of pregnancy. "
"On average, the accelerometer was worn for more than 10 hours per day on 89% of the available days, ranging from 44% to 100%. This percentage was above 80% in all 5 continents of the game. "

13b) For each group, losses and exclusions after randomisation, together with reasons

13b-i) Attrition diagram

Strongly recommended: An attrition diagram (e.g., proportion of participants still logging in or using the intervention/comparator in each group plotted over time, similar to a survival curve) [5] or other figures or tables demonstrating usage/dose/engagement.

	1	2	3	4	5	
subitem not at all important	○	○	○	○	○	essential

Comment on subitem 13b-i)

" Accelerometer-wear was the lowest in the last continent since 4 participants did not use the accelerometer at all because of several reasons (2 holiday in remote area, 1 loss of device, 1 lack of motivation)"
The attrition diagram is presented in the manuscript as figure 3.

Add a subitem under CONSORT item 13b

14a) Dates defining the periods of recruitment and follow-up

14a-i) Indicate if critical "secular events" [1] fell into the study period

Indicate if critical "secular events" [1] fell into the study period, e.g., significant changes in Internet resources available or "changes in computer hardware or Interent delivery resources" [1].

	1	2	3	4	5	
subitem not at all important	○	○	○	○	○	essential

Comment on subitem 14a-i)

There were no critical events in the study period.

Add a subitem under CONSORT item 14a

14b) Why the trial ended or was stopped (early)

(no EHEALTH-specific subitems under CONSORT item 14b)

Comment below to suggest a subitem

not applicable.

15) A table showing baseline demographic and clinical characteristics for each group

NPT: When applicable, a description of care providers (case volume, qualification, expertise, etc.) and centers (volume) in each group

15-i) Report demographics associated with digital divide issues

In ehealth trials it is particularly important to report demographics associated with digital divide issues, such as age, education, gender, social-economic status, computer/Internet/ehealth literacy of the participants, if known.

	1	2	3	4	5	
subitem not at all important	○	○	○	○	○	essential

Comment on subitem 15-i)

Is shown in table 1 of the manuscript.

Add a subitem under CONSORT item 15

16) For each group, number of participants (denominator) included in each analysis and whether the analysis was by original assigned groups

16-i) Report multiple “denominators” and provide definitions

Report multiple “denominators” and provide definitions: Report N’s (and effect sizes) “across a range of study participation [and use] thresholds” [1], e.g., N exposed, N consented, N used more than x times, N used more than y weeks, N participants “used” the intervention/comparator at specific pre-defined time points of interest (in absolute and relative numbers per group). Always clearly define “use” of the intervention.

	1	2	3	4	5	
subitem not at all important	○	○	○	○	○	essential

Comment on subitem 16-i)

One participants was excluded from analyses because of pregnancy.

16-ii) Primary analysis should be intent-to-treat

Primary analysis should be intent-to-treat, secondary analyses could include comparing only “users”, with the appropriate caveats that this is no longer a randomized sample (see 18-i).

	1	2	3	4	5	
subitem not at all important	○	○	○	○	○	essential

Comment on subitem 16-ii)

Table 3 shows our multivariable association between baseline characteristics and program-usage, and our primary outcome 'physical activity' in 51 participants, showing separate models using linear mixed models.

Add a subitem under CONSORT item 16

17a) For each primary and secondary outcome, results for each group, and the estimated effect size and its precision (such as 95% confidence interval)

17a-i) Presentation of process outcomes such as metrics of use and intensity of use

In addition to primary/secondary (clinical) outcomes, the presentation of process outcomes such as metrics of use and intensity of use (dose, exposure) and their operational definitions is critical. This does not only refer to metrics of attrition (13-b) (often a binary variable), but also to more continuous exposure metrics such as “average session length”. These must be accompanied by a technical description how a metric like a “session” is defined (e.g., timeout after idle time) [1] (report under item 6a).

	1	2	3	4	5	
subitem not at all important	○	○	○	○	○	essential

Comment on subitem 17a-i)

In addition to primary and secondary outcomes, measures of compliance and engagement are described and also the number of logins to the program and the number of messages posted at the web-based forum.

Add a subitem under CONSORT item 17a

17b) For binary outcomes, presentation of both absolute and relative effect sizes is recommended

(no EHEALTH-specific subitems under CONSORT item 17b)

Comment below to suggest a subitem

Engagement is presented as a binary outcome, and is analyzed being a determinant of the primary and secondary outcomes. Beta's and 95% confidence intervals are presented.

18) Results of any other analyses performed, including subgroup analyses and adjusted analyses, distinguishing pre-specified from exploratory

18-i) Subgroup analysis of comparing only users

A subgroup analysis of comparing only users is not uncommon in ehealth trials, but if done, it must be stressed that this is a self-selected sample and no longer an unbiased sample from a randomized trial (see 16-iii).

	1	2	3	4	5	
subitem not at all important	○	○	○	○	○	essential

Comment on subitem 18-i)

no other analyses were performed.

Add a subitem under CONSORT item 18

19) All important harms or unintended effects in each group

(for specific guidance see CONSORT for harms)

19-i) Include privacy breaches, technical problems

Include privacy breaches, technical problems. This does not only include physical “harm” to participants, but also incidents such as perceived or real privacy breaches [1], technical problems, and other unexpected/unintended incidents. “Unintended effects” also includes unintended positive effects [2].

	1	2	3	4	5	
subitem not at all important	○	○	○	○	○	essential

Comment on subitem 19-i)

these did not occur

19-ii) Include qualitative feedback from participants or observations from staff/researchers

Include qualitative feedback from participants or observations from staff/researchers, if available, on strengths and shortcomings of the application, especially if they point to unintended/unexpected effects or uses. This includes (if available) reasons for why people did or did not use the application as intended by the developers.

	1	2	3	4	5	
subitem not at all important	○	○	○	○	○	essential

Comment on subitem 19-ii)

This was not subject of study for this manuscript, but users will be asked to join a focus group as part of future upgrading of this intervention.

Add a subitem under CONSORT item 19

DISCUSSION

22) Interpretation consistent with results, balancing benefits and harms, and considering other relevant evidence

NPT: In addition, take into account the choice of the comparator, lack of or partial blinding, and unequal expertise of care providers or centers in each group

22-i) Restate study questions and summarize the answers suggested by the data [2], starting with primary outcomes and process outcomes (use)

Restate study questions and summarize the answers suggested by the data [2], starting with primary outcomes and process outcomes (use).

	1	2	3	4	5	
subitem not at all important	○	○	○	○	○	essential

Comment on subitem 22-i)

"In this clinical pilot-study in an overweight or obese working population, we evaluated the levels of PA during a web-based gaming intervention using a 3D-accelerometer and we assessed changes in BMI and waist circumference versus baseline. We found that levels of PA remained high during our intervention, and in addition, reductions in BMI and waist circumference were achieved. Key components for success were social interaction by eHealth-teams and the level of [illegible]

22-ii) Highlight unanswered new questions, suggest future research [2]

Highlight unanswered new questions, suggest future research [2].

1	2	3	4	5

subitem not at all important ○ ○ ○ ○ ○ essential

Comment on subitem 22-ii)

-broader implementation of a web-based gaming intervention with focus on eHealth-teams and engagement will be beneficial for overweight and obese individuals, and long-term effects should be studied.
-more research is needed to find out how and by whom social support should be delivered and to predict for whom this could work.

Add a subitem under CONSORT item 22

20) Trial limitations, addressing sources of potential bias, imprecision, and, if relevant, multiplicity of analyses

20-i) Typical limitations in ehealth trials

Typical limitations in ehealth trials: Participants in ehealth trials are rarely blinded. Ehealth trials often look at a multiplicity of outcomes, increasing risk for a Type I error. Discuss biases due to non-use of the intervention/usability issues, biases through informed consent procedures, unexpected events.

	1	2	3	4	5	
subitem not at all important	○	○	○	○	○	essential

Comment on subitem 20-i)

-small number of individuals without a control group. Nevertheless, this compact setting provided enough information to suggest broader implementation along with a follow-up study including more individuals in a randomized controlled setting.
-gaming elements in our intervention were mainly focused on PA. - the follow-up time of half a year is insufficient to determine the effectiveness of weight loss maintenance and to [illegible]

Add a subitem under CONSORT item 20

21) Generalisability (external validity, applicability) of the trial findings

NPT: External validity of the trial findings according to the intervention, comparators, patients, and care providers or centers involved in the trial

21-i) Generalizability to other populations

Generalizability to other populations: In particular, discuss generalizability to a general Internet population, outside of a RCT setting, and general patient population, including applicability of the study results for other organizations [2].

	1	2	3	4	5	
subitem not at all important	○	○	○	○	○	essential

Comment on subitem 21-i)

When proven effective, the prototype can be easily adapted to other target-groups, such as obese adolescents and children, elderly, and in oncologic rehabilitation. "

21-ii) Discuss if there were elements in the RCT that would be different in a routine application setting

Discuss if there were elements in the RCT that would be different in a routine application setting (e.g., prompts/reminders, more human involvement, training sessions or other co-interventions) and what impact the omission of these elements could have on use, adoption, or outcomes if the intervention is applied outside of a RCT setting.

	1	2	3	4	5	
subitem not at all important	○	○	○	○	○	essential

Comment on subitem 21-ii)

not applicable

Add a subitem under CONSORT item 21

OTHER INFORMATION

23) Registration number and name of trial registry

(no EHEALTH-specific subitems under CONSORT item 23)

Comment below to suggest a subitem

the trial was not registered.

24) Where the full trial protocol can be accessed, if available

(no EHEALTH-specific subitems under CONSORT item 24)

Comment below to suggest a subitem

the protocol can be accessed by the occupational health center of the Erasmus Medical Center.

25) Sources of funding and other support (such as supply of drugs), role of funders

(no EHEALTH-specific subitems under CONSORT item 25)

Comment below to suggest a subitem

"The development of the movement-game was funded by SoFoKleS ('Sociaal Fonds Kennissector'), a Dutch fund supporting innovations in academic work settings. The implementation was funded by ErasmusMC. Van Rossum was supported by an ErasmusMC research fellowship"

X27) (not a CONSORT item)

X27-i) State the "relation of the study team towards the system being evaluated"

In addition to the usual declaration of interests (financial or otherwise), also state the "relation of the study team towards the system being evaluated" [2], i.e., state if the authors/evaluators are distinct from or identical with the developers/sponsors of the intervention.

	1	2	3	4	5	
subitem not at all important	○	○	○	○	○	essential

Comment on subitem X27-i)

"Conflicts of interest
The author T. Kouwenhoven-Pasmooij is the developer of the movement-game. "
The pilot study was performed by the occupational health center of the Erasmus Medical Center.

Add a subitem under item X27

Last question

Do you want to become involved in the writing committee working on the elaboration document? If yes, please provide the subitems you wish to elaborate on

e.g., 3b-i, 5

Verzenden

Verzend nooit wachtwoorden via Google Formulieren.

Mogelijk gemaakt door

Deze inhoud is niet gemaakt of goedgekeurd door Google.

Misbruik rapporteren - Servicevoorwaarden - Aanvullende voorwaarden
