# Supplementary figures and images for: A Blended Web-Based Gaming Intervention on Changes in Physical Activity for Overweight and Obese Employees: Influence and Usage in an Experimental Pilot Study
Source: JMIR Serious Games. 2017 Apr 3;5(2):e6. doi: 10.2196/games.6421 (PMC5394263; doi:10.2196/games.6421)

## Slide 1
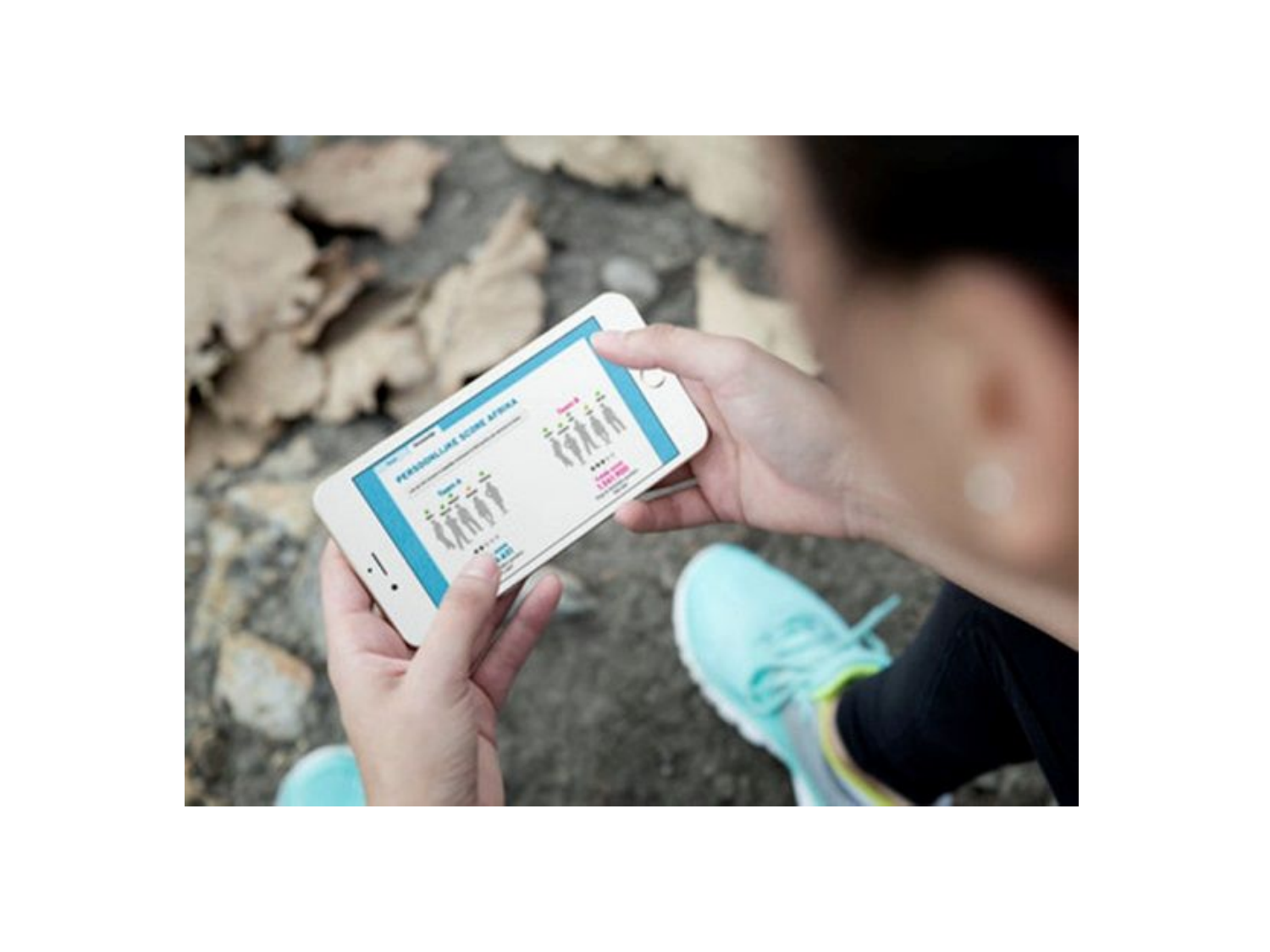

## Slide 2
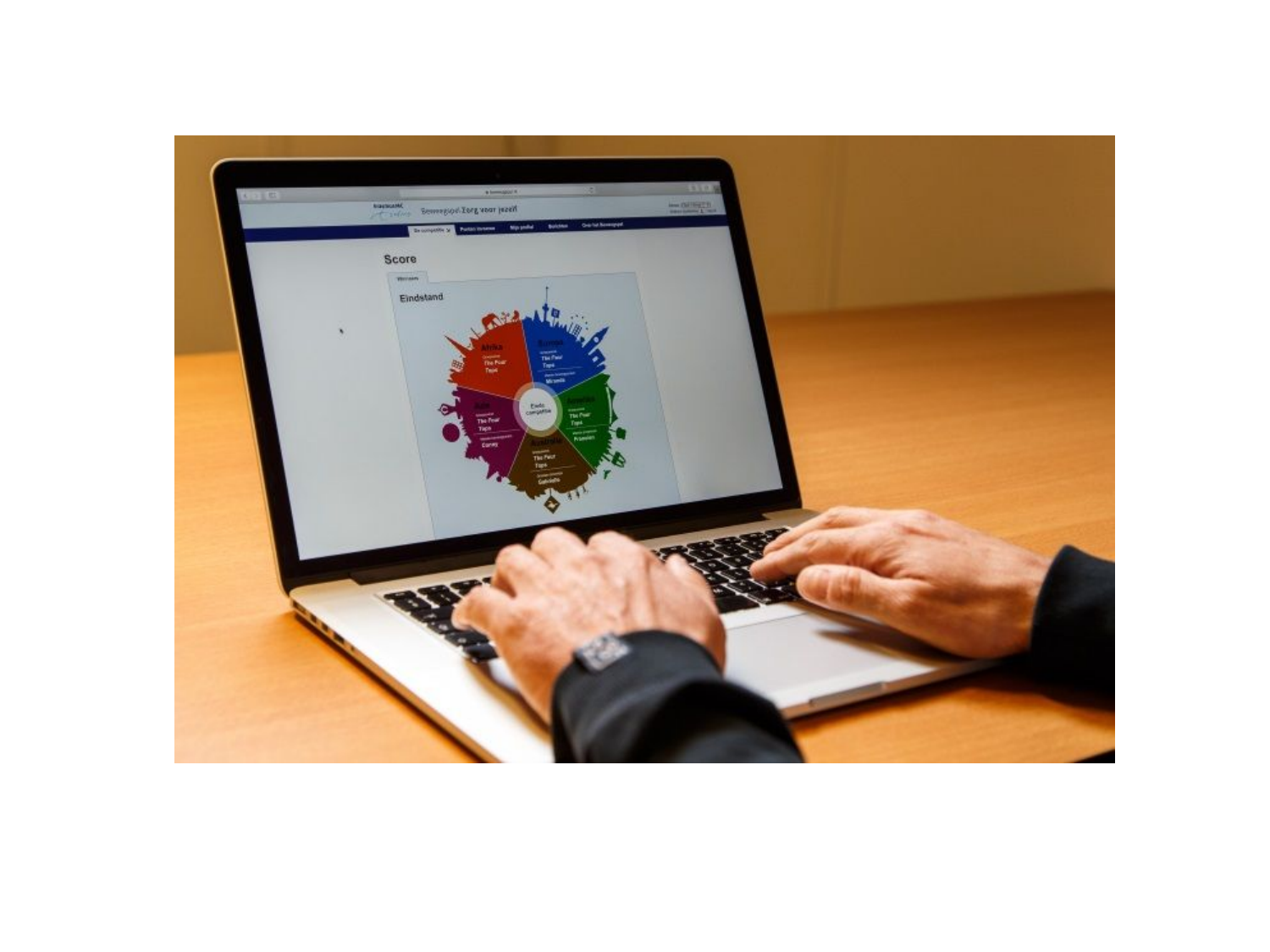

## Slide 3
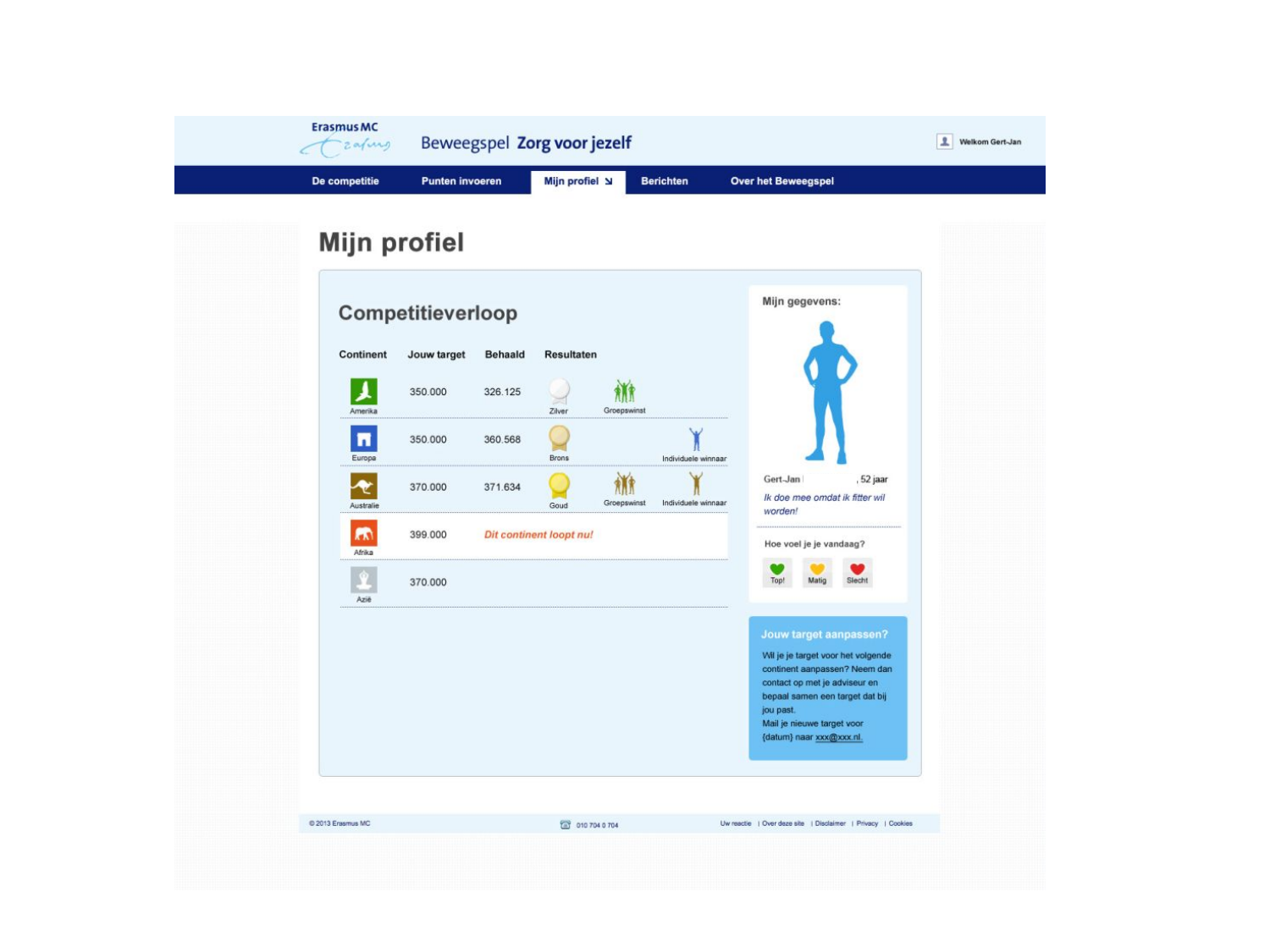

## Slide 4
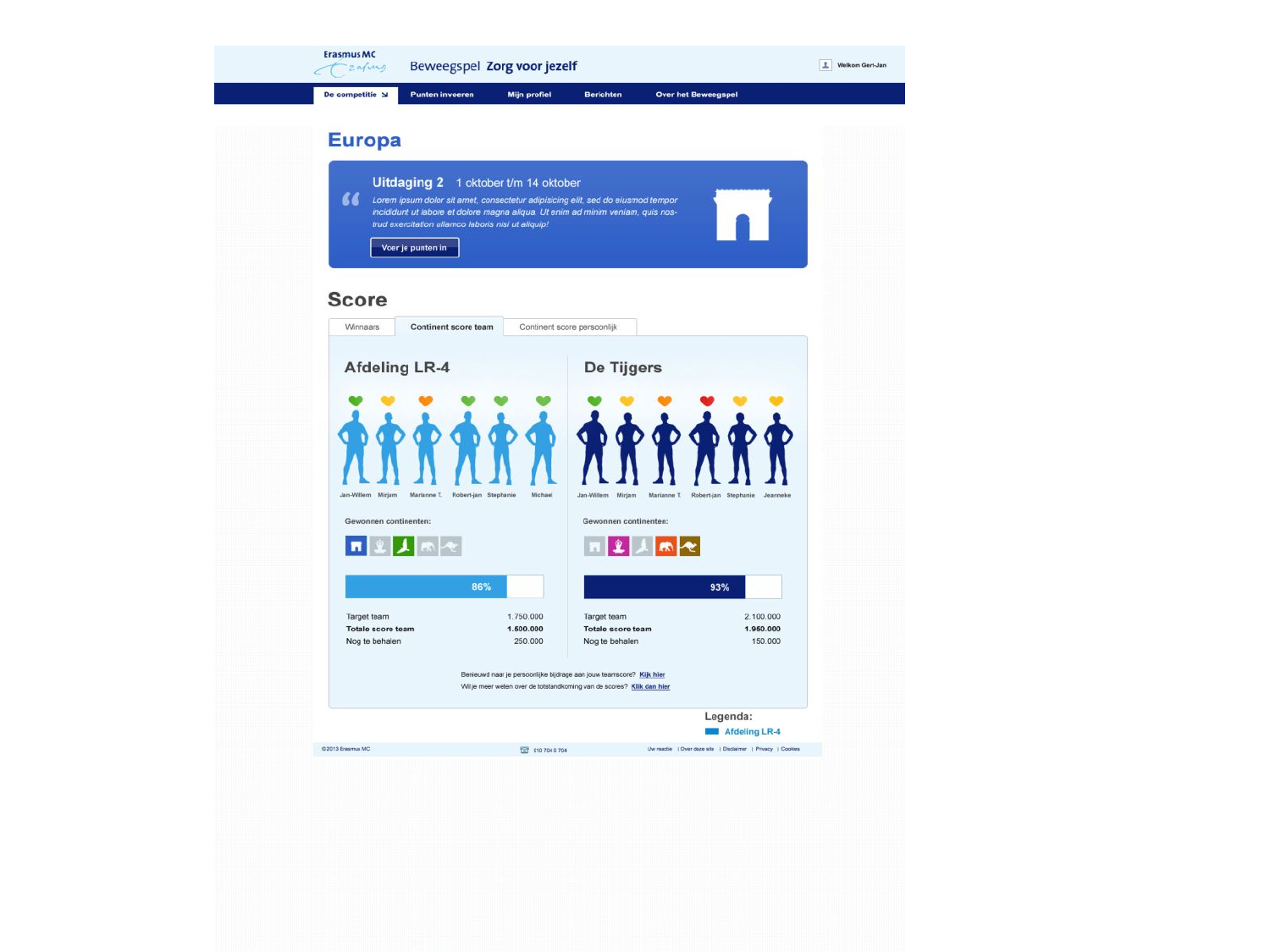

## Slide 5
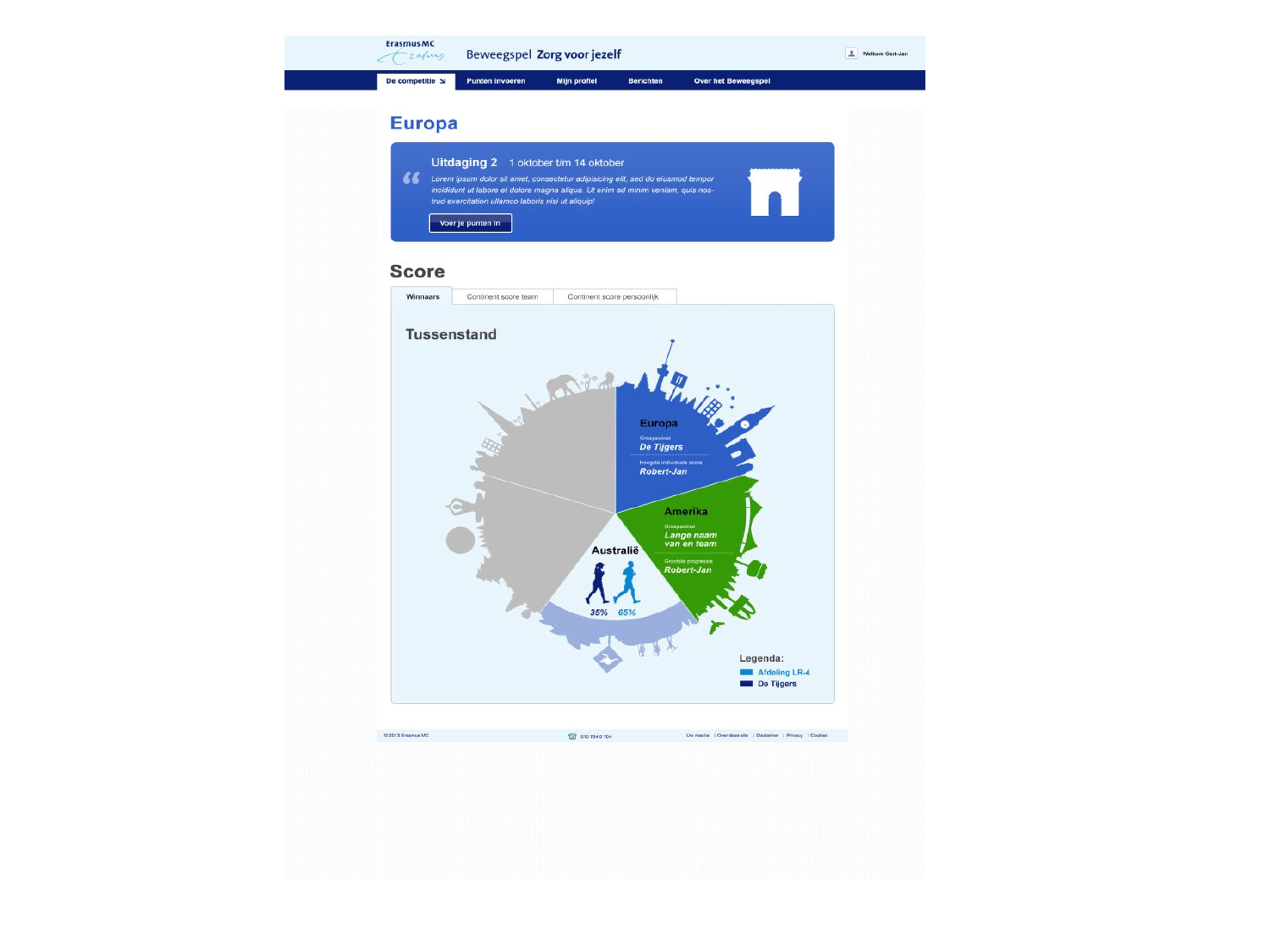

Supplement: Multimedia Appendix 2 [file games_v5i2e6_app2.pptx]
