# Supplementary material for: A Blended Web-Based Gaming Intervention on Changes in Physical Activity for Overweight and Obese Employees: Influence and Usage in an Experimental Pilot Study
Source: JMIR Serious Games. 2017 Apr 3;5(2):e6. doi: 10.2196/games.6421 (PMC5394263; doi:10.2196/games.6421)
Supplement: Multimedia Appendix 3 [file games_v5i2e6_app3.pdf]

Characteristics		Δ BMI[a,b] kg/m^2		Δ Waist[b] cm	
		B (95% CI)	*P*-value[c]	B (95% CI)	*P*-value[c]
Sex					
	Women<br>Men	Reference<br>−0.29 (−1.43 to 0.85)	.61	Reference<br>−0.44 (−5.09 to 4.22)	.85
Age (years)		−0.03 (−0.08 to 0.02)	.27	−0.12 (−0.35 to 0.10)	.30
Educational level					
	Low or medium<br>High	Reference<br>0.28 (−0.74 to 1.31)	.58	Reference<br>3.00 (−1.00 to 7.00)	.14
BMI (kg/m^2)		0.12 (0.04 to 0.20)	.01	0.42 (0.07 to 0.76)	.02
Waist circumference (cm)		0.04 (0.00 to 0.07)	.03	0.22 (0.09 to 0.36)	.01
Work (hrs / week)		0.008 (−0.06 to 0.08)	.82	0.18 (−0.08 to 0.45)	.17
Shift work	Yes<br>No	Reference<br>0.14 (−0.95 to 1.25)	.80	Reference<br>−3.44 (−8.11 to 1.23)	.15
**Characteristics of program-usage**					
eHealth-team					
	1	−0.29 (−2.30 to 1.73)	.78	0.16 (−0.74 to 7.38)	.97
	2	1.23 (−0.95 to 3.42)	.26	4.16 (−3.61 to 11.93)	.29
	3	1.39 (−0.12 to 2.90)	.08	7.16 (−0.06 to 14.38)	.05
	4	−0.31 (−1.47 to 2.93)	.70	−0.09 (−7.86 to 7.68)	.98
	5	0.62 (−0.82 to 1.29)	.40	−1.90 (−7.37 to 3.60)	.49
	6	−0.51 (−2.01 to 1.00)	.50	−2.49 (−9.02 to 4.05)	.45
	7	1.24 (−0.26 to 2.74)	.10	1.80 (−4.74 to 8.34)	.58
	8	Reference		Reference	
Compliance	Accelerometer-wear[d]	0.02 (−0.00 to 0.04)	.07	0.04 (−0.04 to 0.132)	.32
Engagement[e]	≤3 times<br>4 or 5 times	Reference<br>1.05 (0.17;1.93)	.02	Reference<br>4.80 (1.27;8.33)	.009
MVPA[f,g]		0.10 (−0.30;0.51)	.61	1.33 (−0.40;3.01)	.129

Number of logins		0.02 (0.01;0.02)	<0.001	0.03 (0.00;0.06)	.045
Number of messages		0.02 (0.00;0.03)	.01	0.09 (0.01;0.18)	.026

[a] BMI: body mass index.

[b]Δ of outcome = reduction in outcome calculated by measurement at baseline minus measurement at follow-up.

[c]Statistical significance was defined as *P*<.05.

[d]Compliance is expressed as the total number of days with > 10 hours of PA registration, with a maximum of 140 days.

[e]Engagement is expressed as the number of times at least 100% of the target was reached (1-5).

[f]MVPA: moderate to vigorous physical activity.

[g]MVPA is expressed in the average moderate-to-vigorous physical activity in MET-hours per day.
