# Supplementary material for: A Blended Web-Based Gaming Intervention on Changes in Physical Activity for Overweight and Obese Employees: Influence and Usage in an Experimental Pilot Study
Source: JMIR Serious Games. 2017 Apr 3;5(2):e6. doi: 10.2196/games.6421 (PMC5394263; doi:10.2196/games.6421)
Supplement: Multimedia Appendix 4 [file games_v5i2e6_app4.pdf]

Characteristics of program-usage: models			Δ Waist[a] cm		
			Sources of variance SD (95%CI)		B (95% CI)
Model: description	Independent variables (additional)	Categories	Between teams	Between participants	
Model 1: raw			N/A[b]	4.40 (2.30 to 8.40)	N/A
Model 2: baseline characteristics			N/A	3.37 (1.33 to 8.56)	
	Age (years)				−0.09 (−0.31 to 0.14)
	Sex	Women			Reference
		Men			−4.95 (−9.96 to 0.06)
	Waist at baseline				0.27 (0.12 to 0.42)[g]
Model 3: model 2 + eHealth-team			1.79 (0.33 to 9.61)	3.17 (1.23 to 8.20)	
Model 4: model 3 + compliance			1.54(−0.17 to 12.91)	3.19(1.32 to 7.70)	
	Compliance[c]	<85%			Reference
		85-95%			−1.98 (−6.61 to 2.66)
		≥95%			0.80 (−3.41 to 5.01)
Model 5: model 3 + engagement			1.50(−0.24 to 9.52)	2.43(0.44 to 13.30)	
	Engagement[d]	≤3			Reference
		4 or 5			4.44 (0.84 to 8.03)
Model 6: Model 3 + MVPA			1.24(−0.00 to 21.87)	3.02(1.07 to 8.47)	
	MVPA[e,f]				0.20 (−0.16 to 0.55)
Model 7: model 3 + compliance + engagement+ MVPA			1.68(−0.26 to 10.45)	2.70(0.74 to 9.78)	
	Compliance[c]	<85%			Reference

		85%-95%			−1.25 (−5.93 to 3.44)
		≥95%			−0.91 (−5.58 to 3.76)
	Engagement[d]	≤3			Reference
		4 or 5			4.03 (−0.93 to 8.98)
	MVPA[e,f]				0.01 (−0.44 to 0.45)
	Time	0-10 weeks			Reference
		0-23 weeks			−1.69 (−6.14 to 2.75)

[a]Δ of outcome = reduction in outcome calculated by measurement at baseline minus measurement at follow-up.

[b]N/A: not applicable.

[c] Compliance is expressed as percentage of days with > 10 hours of PA registration (accelerometer-wear).

[d]Engagement is expressed as the number of times at least 100% of the target was reached (1-5)

[e]MVPA: moderate to vigorous physical activity

[f]MVPA is expressed as the average moderate-to-vigorous physical activity in MET-hours per day, not log-transformed.

[g]Statistically significant at *P*<.05.
